# The Evolutionarily Conserved Longevity Determinants HCF-1 and SIR-2.1/SIRT1 Collaborate to Regulate DAF-16/FOXO

**DOI:** 10.1371/journal.pgen.1002235

**Published:** 2011-09-01

**Authors:** Gizem Rizki, Terri Naoko Iwata, Ji Li, Christian G. Riedel, Colette Lafontaine Picard, Max Jan, Coleen T. Murphy, Siu Sylvia Lee

**Affiliations:** 1 Department of Molecular Biology and Genetics, Cornell University, Ithaca, New York, United States of America; 2 Department of Molecular Biology and Genetics, Field of Comparative Biomedical Sciences, Cornell University, Ithaca, New York, United States of America; 3 Department of Molecular Biology, Massachusetts General Hospital, Harvard Medical School, Simches Research Center, Boston, Massachusetts, United States of America; 4 Department of Molecular Biology, Princeton University, Princeton, New Jersey, United States of America; University of Washington, United States of America

## Abstract

The conserved DAF-16/FOXO transcription factors and SIR-2.1/SIRT1 deacetylases are critical for diverse biological processes, particularly longevity and stress response; and complex regulation of DAF-16/FOXO by SIR-2.1/SIRT1 is central to appropriate biological outcomes. *Caenorhabditis elegans* Host Cell Factor 1 (HCF-1) is a longevity determinant previously shown to act as a co-repressor of DAF-16. We report here that HCF-1 represents an integral player in the regulatory loop linking SIR-2.1/SIRT1 and DAF-16/FOXO in both worms and mammals. Genetic analyses showed that *hcf-1* acts downstream of *sir-2.1* to influence lifespan and oxidative stress response in *C. elegans*. Gene expression profiling revealed a striking 80% overlap between the DAF-16 target genes responsive to *hcf-1* mutation and *sir-2.1* overexpression. Subsequent GO-term analyses of HCF-1 and SIR-2.1-coregulated DAF-16 targets suggested that HCF-1 and SIR-2.1 together regulate specific aspects of DAF-16-mediated transcription particularly important for aging and stress responses. Analogous to its role in regulating DAF-16/SIR-2.1 target genes in *C. elegans*, the mammalian HCF-1 also repressed the expression of several FOXO/SIRT1 target genes. Protein–protein association studies demonstrated that SIR-2.1/SIRT1 and HCF-1 form protein complexes in worms and mammalian cells, highlighting the conservation of their regulatory relationship. Our findings uncover a conserved interaction between the key longevity determinants SIR-2.1/SIRT1 and HCF-1, and they provide new insights into the complex regulation of FOXO proteins.

## Author Summary

The nematode *C. elegans* has been instrumental in identifying and characterizing genetic components that influence aging. Studies in worms have been successfully extended to complex mammalian organisms allowing for the identification of genetic factors that impact longevity in mammals. DAF-16/FOXO transcription factors are among the best characterized longevity factors, and their increased activity leads to a longer lifespan and improved stress resistance in many organisms. Elucidating how the activities of DAF-16/FOXO are regulated will provide new insights into the basic biology of aging and will aid future therapeutic developments aiming to improve healthy aging and alleviate age-related diseases in humans. We utilized both *C. elegans* and mammalian cell culture systems to dissect the functional and molecular interactions between two important DAF-16 regulators, HCF-1 and SIR-2.1/SIRT1. We demonstrated that HCF-1 and SIR-2.1/SIRT1 physically associate and antagonize each other to properly regulate DAF-16/FOXO-mediated expression of genes important for longevity and stress response. We further showed that the functional relationships among these three proteins are conserved in mammals. Our work implicates HCF-1 as an important player in the regulation of FOXO by SIRT1, and thereby a potential longevity determinant in humans, and prompts further characterization of HCF-1's functions in aging and age-related pathologies.

## Introduction

The Insulin/Insulin-like Growth Factor-1(IGF-1) signaling (IIS) cascade is one of the most highly conserved and best characterized longevity pathways in eukaryotes. When stimulated, the insulin/IGF-1 like receptors initiate a kinase cascade that leads to the phosphorylation, and cytoplasmic retention of the main downstream effectors, Forkhead box, Class O (FOXO) transcription factors. Reduction in IIS signaling leads to the dephosphorylation of FOXO, allowing nuclear translocation and transcriptional activation of FOXO [1], [2]. The *C. elegans* FOXO ortholog DAF-16, as well as the *Drosophila*, mouse, and human FOXO transcription factors are all critical for longevity, metabolism, and stress response [3]–[12], suggesting that the mechanisms underlying FOXOs' ability to affect physiology are highly conserved across species. Indeed, much of our understanding of FOXO regulation comes from studies done on *C. elegans* DAF-16. When activated, DAF-16 selectively regulates the transcription of a large number of genes which cumulatively act to elevate stress resistance, alter metabolic and developmental responses, improve immunity, and extend lifespan [13]–[16]. To integrate many different environmental stimuli and coordinate proper transcriptional responses, DAF-16 activity must be tightly controlled. DAF-16 activity is known to be regulated by post-translational modifications, nuclear/cytoplasmic translocation and association with transcriptional co-regulators. Although necessary for its activation, translocation of DAF-16 into the nucleus is not sufficient to stimulate its transcriptional activity [17]. Association with additional co-factors is also necessary for nuclear DAF-16 activation [18]–[23]. Little is known about the interplay between DAF-16 and its nuclear regulators and how these multiple factors coordinately act on DAF-16 to ensure proper transcriptional outcomes.

SIR-2.1, the *C. elegans* homolog of the yeast NAD+-dependent protein deacetylase Sir2p, is an important DAF-16 co-factor. SIR-2.1 is thought to activate DAF-16 in conferring longevity as well as stress resistance [18], [24], [25]. Heat stress stimulates the physical association of SIR-2.1 with DAF-16 via the scaffolding protein 14-3-3, which promotes the transactivation of DAF-16 through an unknown mechanism [18], [25]. Overexpression of Sir2 homologs in worms, yeast and flies extends lifespan [18], [24], [26], [27], emphasizing the evolutionarily conserved role of Sir2 in longevity determination. In mammals, SIRT1 associates with and directly deacetylates FOXO1, 3, and 4 in a stress-dependent manner [28]–[31]. However, the exact mechanism whereby SIR-2.1/SIRT1 affects DAF-16/FOXO activity and whether additional factors are involved in the regulation of DAF-16/FOXO by SIR-2.1/SIRT1 is not well understood.

Host Cell Factor-1 (HCF-1) belongs to a family of highly conserved HCF proteins and acts as a nuclear co-repressor of DAF-16 [21], [32]. Inactivating *hcf-1* robustly extends lifespan and confers oxidative stress resistance in a *daf-16*-dependent manner in *C. elegans*. In the nucleus, HCF-1 associates with DAF-16 and limits its access to a subset of target gene promoters [21]. *C. elegans* HCF-1 shares high structural homology with two mammalian counterparts, HCF-1 and HCF-2 [32]. Although mammalian HCF-1 has been studied extensively, HCF-2 functions remain largely unknown. Mammalian HCF-1 was originally identified as a binding partner of the Herpes Simplex Virus VP16 transcription factor [33]. Apart from VP16, HCF-1 has been shown to associate with a number of transcription factors to stimulate or repress their transactivation properties [34]–[39]. HCF-1 is an important regulator of cellular proliferation as it promotes progression through multiple phases of the cell cycle via assembling transcriptional complexes to modulate E2F transcription factor activities [38], [40]. Whether mammalian HCF proteins function as conserved FOXO regulators has yet to be determined.

In this study, we sought to examine whether the two conserved DAF-16/FOXO nuclear regulators, HCF-1 and SIR-2.1/SIRT1, functionally interact in worms and whether this interaction is conserved in mammals. We found that *hcf-1* acts downstream of *sir-2.1* to regulate *daf-16* and thereby modulates lifespan and oxidative stress response in *C. elegans*. We showed that HCF-1 and SIR-2.1 regulate a common subset of DAF-16 target genes important for ensuring longevity and stress response. Furthermore, we demonstrated that mammalian HCF-1 affects the expression of several SIRT1/FOXO transcriptional targets and physically associates with both FOXO3 and SIRT1. Our findings uncover a new regulatory mechanism between the critical longevity determinants DAF-16/FOXO and SIR-2.1/SIRT1, and implicate an important role of HCF-1 in aging and age-related diseases in diverse organisms.

## Results

### *C. elegans hcf-1* acts downstream of *sir-2.1* to modulate longevity and oxidative stress responses

**Figure 1:**
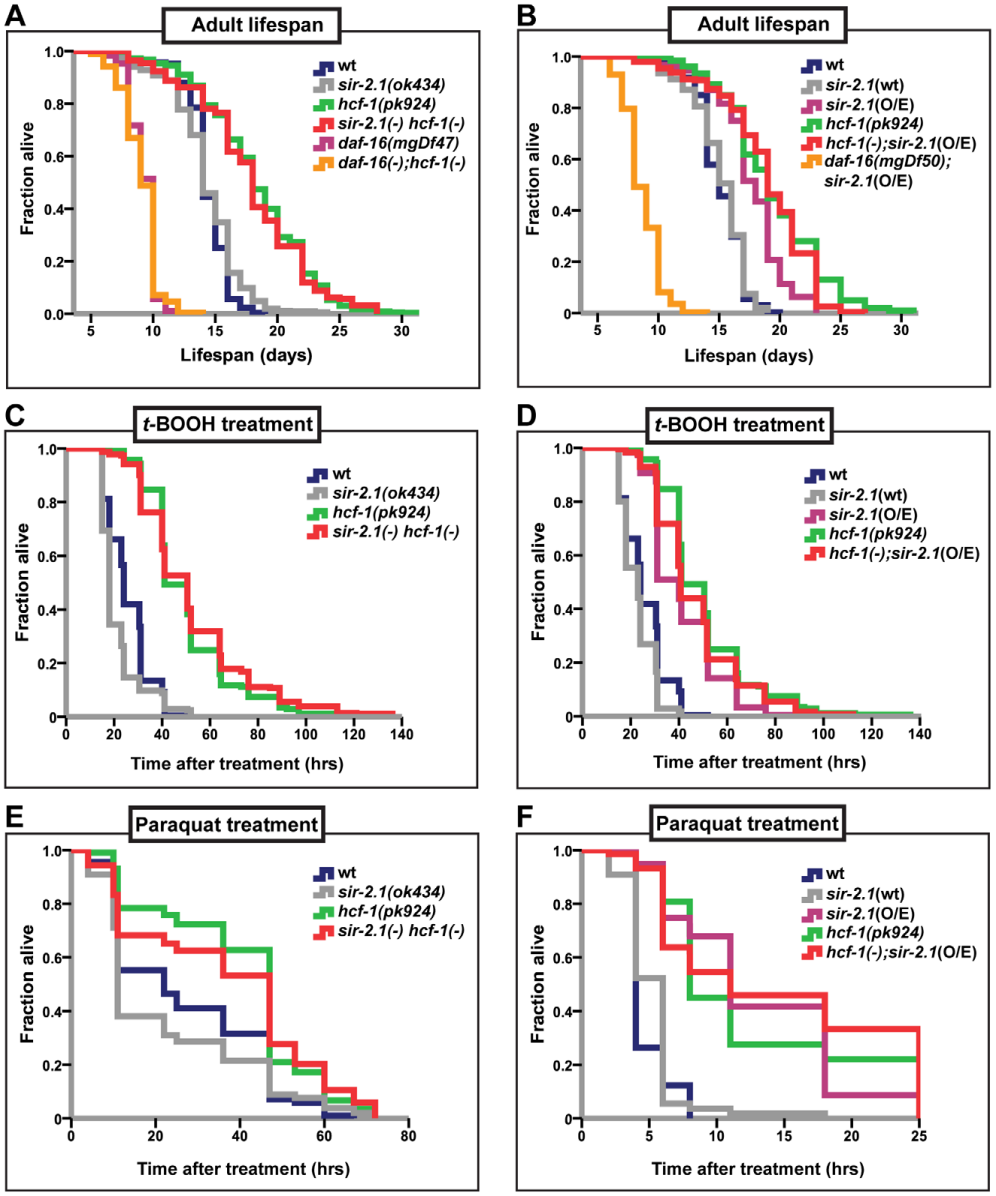
*hcf-1* acts downstream of *sir-2.1* to modulate lifespan and oxidative stress response. (A–B) Lifespans of synchronized adult populations of indicated genotypes. (A) Data pooled from four independent experiments are plotted. One of four *sir-2.1(ok434) hcf-1(pk924)* lines is shown. (See Table S1A). (B) Pooled data from three independent experiments are displayed. One of five *hcf-1(pk924);pkIs1642[sir-2.1*(O/E)*]* lines is shown (See Table S1B). (C–F) Oxidative stress response of adult worms. (C–D) Day one adult worms were exposed to 6 mM *t*-BOOH on plates and their survival monitored through time. The survival curves represent pooled data from two independent experiments. (E–F) Day two adult worms were exposed to 150 mM (E) or 200 mM (F) paraquat in M9 buffer and their survival monitored through time. Survival curves are generated using pooled data from two independent experiments (E) or data from one of two representative experiments (F). See Table S1A–S1F for statistics and Figure S1C–S1F for linear mixed model analysis plots. All lifespan and stress experiments were carried out at 25°C. Quantitative data and statistical analyses are displayed in Table S1A–S1D.

In *C. elegans*, inactivation of *hcf-1* results in a robust lifespan extension, as well as improved survival upon exposure to oxidative stress, in a manner dependent on *daf-16*. In its role in longevity and stress response, HCF-1 inhibits DAF-16 activity by physically associating with DAF-16 and diminishing DAF-16 localization to a subset of downstream target promoters [21]. In the context of cell cycle progression, mammalian HCF-1 is known to regulate the activities of various transcription factors by promoting the formation of transcriptional regulatory complexes [39], [41]. We reasoned that HCF-1 in *C. elegans* may function similarly and, in conjunction with other transcriptional regulators, act to fine tune DAF-16 activity. As SIR-2.1 is a well-known, evolutionarily conserved longevity determinant that activates DAF-16 [18], we explored whether HCF-1 and SIR-2.1 functionally interact to regulate DAF-16. As a first step, we examined the putative functional connection between *hcf-1* and *sir-2.1* in lifespan modulation by performing genetic analyses. We compared the lifespan of *hcf-1(pk924)* and *sir-2.1(ok434)* single mutants to that of *sir-2.1(ok434) hcf-1(pk924)* double mutants. Both *hcf-1* and *sir-2.1* alleles used in this analysis are putative null mutants [21], [42]. As previously described, *hcf-1(pk924)* mutant worms displayed a mean lifespan >20% longer than that of wild type and the *hcf-1(pk924)* long-lived phenotype was fully suppressed by *daf-16(mgDf47)* mutation (Figure 1A and [21]). *sir-2.1(ok434)* mutants exhibited lifespan similar to that of wild-type worms and always substantially shorter than that of *hcf-1(pk924)* (Figure 1A; Table S1A). We found that all four independent lines of the double mutants exhibited lifespans similar to that of *hcf-1(pk924)* single mutant worms (Figure 1A, Table S1A), suggesting that *sir-2.1* is not required for *hcf-1(pk924)* mutation to extend lifespan. Our genetic data suggest two possibilities: one is that *hcf-1* and *sir-2.1* may work independently and that *sir-2.1* inactivation does not affect *hcf-1(pk924)* mutant longevity. On the other hand, since the lifespan of the double mutant is similar to that of *hcf-1(pk924)* single mutant, *hcf-1* may act downstream of *sir-2.1*. To distinguish between these two possibilities, we examined the effect of overexpressing *sir-2.1* in worms harboring the *hcf-1* mutation. In *C. elegans*, overexpressing *sir-2.1* confers a lifespan extension phenotype that is dependent on *daf-16* [18], [24]. We reasoned that if *hcf-1* and *sir-2.1* work independently, then combining *hcf-1* inactivation with *sir-2.1* overexpression should further increase lifespan. By contrast, if *hcf-1* and *sir-2.1* work in the same pathway, and *hcf-1* is genetically downstream of *sir-2.1*, then overexpression of *sir-2.1* should not cause further lifespan extension in *hcf-1(pk924)* mutants. To examine this, we utilized the long-lived, low-copy *sir-2.1* overexpressor strain NL3909 *pkIs1642 [unc-119 sir-2.1]* (*pkIs1642[sir-2.1*(O/E)*]*) [18], [43] to generate *hcf-1(pk924);pkIs1642[sir-2.1*(O/E)*]* strains. As a control, we outcrossed the *pkIs1642* strain and showed that it continues to extend lifespan compared to its transgenic control NL3908 *pkIs1641 [unc-119]* (*pkIs1641[sir-2.1*(wt)*]*) under our assaying conditions (Figure S2A; Table S1G). Furthermore, we knocked-down *sir-2.1* in the *pkIs1642* strain to show that the lifespan increase is indeed dependent on *sir-2.1* (Figure S2B–S2D; Table S1H). *hcf-1(pk924)* and *pkIs1642[sir-2.1*(O/E)*]* worms lived longer than N2 wild type or *pkIs1641[sir-2.1*(wt)*]* transgenic controls by 28% and 17%, respectively (Figure 1B; Table S1B, S1G). Interestingly, the *hcf-1(pk924);pkIs1642[sir-2.1*(O/E)*]*) worms exhibited a lifespan very similar to, or in some cases shorter than, that of *hcf-1(pk924)* mutants (Figure 1B; Table S1B). However, in none of the *hcf-1(pk924);pkIs1642[sir-2.1*(O/E)*]*) isolates generated did we observe a lifespan longer than that of *hcf-1(pk924)* mutants (Table S1B). These data support the hypothesis that *hcf-1* acts in the same genetic pathway as *sir-2.1*. Considering our previous findings that *hcf-1* can robustly extend the lifespans of long-lived insulin signaling and germline proliferation mutants [21], our current observation that overexpression of *sir-2.1* cannot further enhance longevity in worms lacking *hcf-1* indicates that the genetic interaction between *hcf-1(-)* and *sir-2.1*(O/E) is specific.

In addition to their lifespan effects, both HCF-1 and SIR-2.1 regulate the ability of DAF-16 to respond to a variety of environmental stress cues. Adult *hcf-1(pk924)* mutant worms are resistant to oxidative- and heavy metal-stress [21]. Likewise, *sir-2.1* overexpression is protective against exposure to oxidative as well as heat stress, while *sir-2.1* mutation increases sensitivity to oxidative, heat, and UV-induced environmental insults [18], [42]. To further investigate the genetic relationship between *hcf-1* and *sir-2.1*, we analyzed the response of *sir-2.1(ok434) hcf-1(pk924)* double mutants and *hcf-1(pk924)*;*pkIs1642[sir-2.1*(O/E)*]*) worms to treatment with two oxidative-stress inducing agents, paraquat and *tert*-Butyl hydroperoxide (*t*-BOOH). Paraquat induces cellular damage by elevating intracellular superoxide levels [44], and *t*-BOOH damages cellular lipids and proteins through peroxidation [45]. Under the paraquat or *t*-BOOH conditions where *sir-2.1(ok434)* mutants were sensitive and *hcf-1(pk924)* worms resistant to the treatments, *sir-2.1(ok434) hcf-1(pk924)* worms survived the paraquat or *t*-BOOH exposure as well as *hcf-1(pk924)* single mutants did, and were significantly more resistant than N2 or *sir-2.1(ok434)* worms (Figure 1C, 1E; Figure S1A, S1C; Table S1C, S1E). Furthermore, overexpressing *sir-2.1* in *hcf-1(pk924)* mutants did not further enhance the paraquat or *t*-BOOH-resistance of *hcf-1(pk924)* worms (Figure 1D, 1F; Figure S1B, S1D; Table S1D, S1F). Overall, our observations are consistent with a model in which *hcf-1* acts downstream of *sir-2.1* to modulate longevity and oxidative stress responses in *C. elegans*.

### 14-3-3 proteins are required for lifespan extension in worms carrying the *hcf-1* mutation

**Figure 2:**
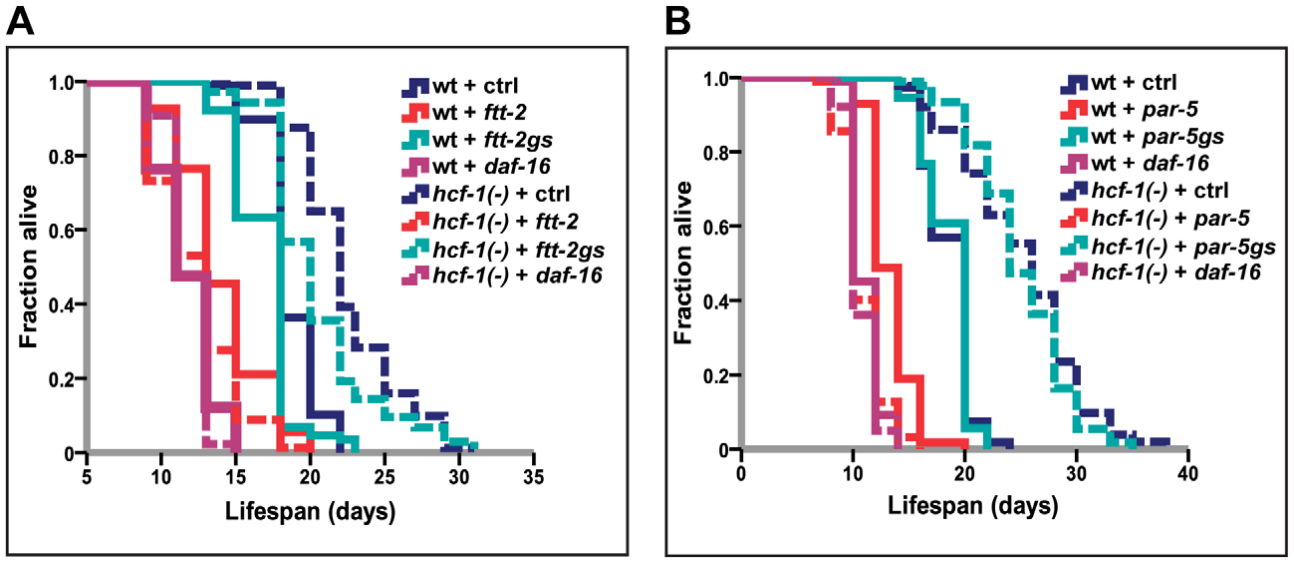
*14-3-3* are required for lifespan extension conferred by *hcf-1(pk924)* mutation. (A–B) Worms were grown on vector, *daf-16*, *ftt-2* (Ahringer - contains multiple stretches of identical sequences to *par-5*), *par-5* (Ahringer - contains overlapping sequences with *ftt-2*) [47], *ftt-2gs* (gene specific RNAi targeting 3′ UTR of *ftt-2*), or *par-5gs* (gene-specific RNAi targeting 3′ UTR of *par-5*) [47] from egglay until the end of life. The lifespan experiments were carried out at 20°C. Quantitative data and statistical analyses are included in Table S2A, S2B.

In *C. elegans*, 14-3-3 proteins are required for lifespan extension and stress resistance conferred by extra copies of *sir-2.1*, as well as for facilitating the association of SIR-2.1 and DAF-16 [18], [25]. Our findings that *hcf-1* and *sir-2.1* act together to regulate *daf-16* raise the possibility that *hcf-1* may also functionally interact with *14-3-3*. To address this question, we examined the genetic relationship between *hcf-1* and *14-3-3* in lifespan. The 14-3-3 homologs in *C. elegans* are encoded by two highly similar genes *ftt-2* and *par-5*, which share ∼80% sequence identity [46]. RNAi constructs targeting the coding sequences of *ftt-2* and *par-5* are not specific and will knockdown both genes, whereas RNAi constructs targeting the 3′ UTR of each are gene-specific (Figure S4A and [47]). We found that knocking down either *ftt-2* or *par-5* alone did not substantially reduce *hcf-1(pk924)* lifespan, yet simultaneously diminishing the function of both genes through the non-specific RNAi completely abrogated the longevity effect of *hcf-1* inactivation (Figure 2A, 2B; Table S2A, S2B). The RNAi data are corroborated by our findings that a null mutation of *ftt-2*, *n4426*, was only able to slightly decrease the lifespan of *hcf-1* mutants (Figure S3D; Table S2D). Therefore, we conclude that both *14-3-3* genes are necessary for the longevity increase conferred by *hcf-1* mutation and likely act downstream of *hcf-1*.

### *hcf-1* and *sir-2.1* co-regulate a specific subset of DAF-16 transcriptional targets important for longevity, cellular detoxification, and fatty acid/lipid/amino acid metabolism

DAF-16 responds to different upstream stimuli by selectively activating and repressing groups of target genes, and hence ensuring appropriate responses to specific signals [14]–[16]. We previously proposed that *C. elegans* HCF-1 acts as a specificity factor for DAF-16 and negatively regulates DAF-16 on a select set of its target genes [21]. Similarly, *C. elegans* SIR-2.1 is thought to promote DAF-16 regulation of a subset of transcriptional targets [18]. As our genetic data suggest that *hcf-1* and *sir-2.1* act in the same genetic pathway to modulate longevity in a *daf-16*-dependent manner, we hypothesized that *hcf-1* inactivation and *sir-2.1* overexpression would have similar effects on DAF-16-mediated transcription. To test this hypothesis, we compared the *daf-16-*dependent global transcriptional changes occurring in the long-lived *hcf-1(pk924)* mutant to those occurring in the long-lived *sir-2.1* overexpressor strain.

**Figure 3:**
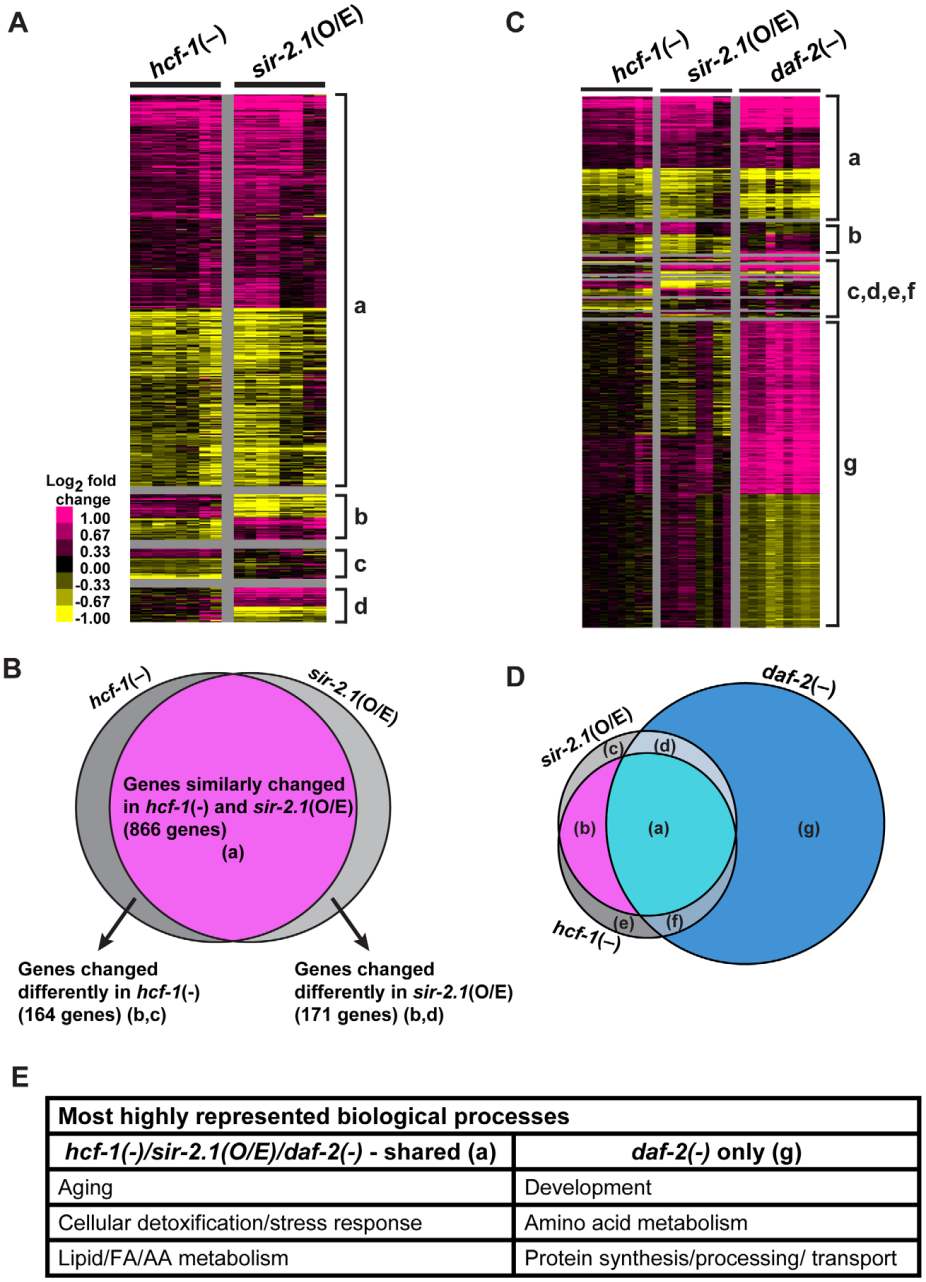
*hcf-1* inactivation and *sir-2.1* overexpression similarly affect a specific subset of *daf-16* downstream target genes. (A–D) Heat maps representing the expression patterns of differentially expressed genes identified by Significance Analysis of Microarrays (SAM) and Venn diagrams showing the overlap between different datasets. For heat maps, each column represents a biological replicate and each row is a gene. Pink = upregulated, Yellow = downregulated, Black = not changed. (A) Heat maps comparing *hcf-1(-)* and *sir-2.1*(O/E) arrays. Gene clusters are categorized as: (a) = Genes similarly changed in *hcf-1(-)* and *sir-2.1*(O/E) (866), (b) = genes oppositely changed in *hcf-1(-)* and *sir-2.1*(O/E) (98), (c) = genes uniquely changed in *hcf-1(-)* (66), (d) = genes uniquely changed in *sir-2.1*(O/E) (73). (B) Venn diagram summarizing overlap in (A). (C) Heat maps comparing *hcf-1(-)*, *sir-2.1*(O/E), and *daf-2(-)* arrays. Genes are clustered as (a) = similarly expressed in all 3 profiles (693), (b) = similar in only *hcf-1(-)* and *sir-2.1*(O/E) (173), (c) = uniquely changed in *sir-2.1*(O/E) (130), (d) = similar in only *sir-2.1*(O/E) and *daf-2(-)* (26), (e) = uniquely changed in *hcf-1(-)* (140), (f) = similar in only *hcf-1(-)* and *daf-2(-)* (46), (g) = uniquely changed in *daf-2(-)* (1750) (See also Table S3). (D) Venn diagram summarizing overlaps in (C). (E) Most highly enriched GO terms (See also Table S4A, S4B) are summarized based on general biological process.

We identified the genes whose expression was changed in *hcf-1(pk924)* mutants in a *daf-16-*dependent manner by comparing the expression profiles of synchronized *hcf-1(pk924)* mutants to those of *daf-16(mgDf47);hcf-1(pk924)* double mutants using Agilent *C. elegans* gene expression microarrays. In addition, to pinpoint the genes that are responsive to the *hcf-1(pk924)* mutation, instead of those that show expression changes simply due to *daf-16* deletion, we focused on genes that showed a similar trend of expression change both in the *hcf-1(pk924)* vs. N2 and *hcf-1(pk924)* vs. *daf-16(mgDf47);hcf-1(pk924)* comparisons (henceforth referred to as *hcf-1(-)* profile) (Data are available at NCBI Gene Expression Omnibus, accession number GSE30725). Likewise, the genes which were differentially regulated by DAF-16 in response to *sir-2.1* overexpression were identified by comparing the strains *pkIs1642[sir-2.1*(O/E)*]* to *daf-16(mgDf50);pkIs1642[sir-2.1*(O/E)*]* and *pkIs1642 [sir-2.1*(O/E)*]* to its transgenic control *pkIs1641[sir-2.1*(wt)*]* (henceforth referred to as *sir-2.1*(O/E) profile). To identify the genes that show consistent and significant expression changes across the independent biological replicates of *hcf-1(-)* or *sir-2.1*(O/E), we used Significance Analysis of Microarrays (SAM) [48] with a stringent criteria of expected false discovery rate (FDR) set at 0%. SAM analysis identified 1,032 significantly affected genes in *hcf-1(-)* and 1,042 genes in *sir-2.1*(O/E) (Figure 3A; Table S3). Next, we compared the two datasets to determine the extent of overlap. Strikingly, we found 866 genes (473 upregulated and 390 downregulated) whose expression changed similarly in *hcf-1(-)* and *sir-2.1*(O/E) profiles, suggesting that the vast majority (>80%) of the genes regulated by DAF-16 in response to *hcf-1* inactivation or *sir-2.1* activation are shared (Figure 3B). Of the genes that were expressed in a dissimilar manner between *hcf-1(-)* and *sir-2.1*(O/E) profiles, ∼10% displayed an opposite expression change and ∼10% were unique to either *hcf-1(-)* or *sir-2.1*(O/E) (Figure 3A, 3B). The finding that the transcriptional outcomes conferred by DAF-16 in response to *hcf-1* mutation or *sir-2.1* overexpression are largely similar corroborates our genetic data suggesting that SIR-2.1 and HCF-1 act in the same pathway to regulate DAF-16.

In addition to being regulated by SIR-2.1 and HCF-1, DAF-16 activity is also controlled by the insulin/IGF-1 signaling (IIS) pathway. In response to reduced IIS, DAF-16 translocates into the nucleus and regulates the expression of a large number of genes that together contribute to the diverse functions of IIS, including the regulation of development, metabolism, stress response, and longevity [14]–[16]. To determine how the *hcf-1*- and *sir-2.1*-responsive DAF-16- target genes compare with the IIS-responsive DAF-16 targets, we further compared the *hcf-1(-)* and *sir-2.*1(O/E) profiles to that of the *daf-2(-)* profile (microarray data from *daf-2(e1370) vs. daf-16(mgDf50);daf-2(e1370)* [49]). Interestingly, expression of the majority of the shared *hcf-1(-)*/*sir-2.1*(O/E)-regulated genes (693/866 = 80%) were also changed in *daf-2(-)* in the same direction, yet this represented only a fraction of all *daf-2(-)-*induced changes (693/2515 = 28%) (Figure 3C, 3D). This indicates that, among a large number of potential DAF-16 targets, *hcf-1* and *sir-2.1* converge to co-regulate a distinct subset of these genes. Our findings from the microarray comparisons support the model that HCF-1 and SIR-2.1 antagonize each other to control a particular aspect of the DAF-16-regulated transcriptional program.

To examine the biological processes that can be carried out by genes affected by *hcf-1(-)* and *sir-2.1*(O/E), we queried their Gene Ontology (GO) terms using Database for Annotation, Visualization, and Integrated Discovery (DAVID) [50]. We focused on the GO term categories most significantly enriched in our dataset compared to the *C. elegans* genome. Our analyses revealed that for the DAF-16 target genes co-regulated by HCF-1/SIR-2.1, GO terms for aging, cellular detoxification (in particular phase 1 & 2 detoxification) and stress response were highly overrepresented among both the upregulated and downregulated genes (Figure 3E; Table S4) [51], [52]. To test whether the DAF-16 targets that are co-regulated by HCF-1/SIR-2.1/DAF-2 might participate in biological functions distinct from the targets uniquely regulated by DAF-2 (and not affected by HCF-1/SIR-2.1), we compared the GO terms represented in the *hcf-1(-)*/*sir-2.*1(O/E)-shared genes to those in *daf-2(-)*. Among the genes induced by DAF-16, the most prominent functional categories represented in the *hcf-1(-)/sir-2.1*(O/E)/*daf-2(-)-*overlapping set were very similar to those in the *hcf-1(-)/sir-2.1*(O/E)-co-regulated set (i.e. aging, detoxification, stress response) (Figure 3E; Table S4). By contrast, the DAF-16 target genes that are uniquely upregulated in *daf-*2*(-)* are enriched for GO categories for developmental, metabolic (amino acid anabolism/catabolism) and cellular ion transport processes (Figure 3E; Table S4A). Among the genes repressed by DAF-16, the *hcf-1(-), sir-2.*1(O/E) and *daf-2(-)* overlapping set is also enriched with GO terms in aging and stress responses, as well as a new category in fatty acid/lipid/amino acid metabolic processes. Interestingly, the *daf-2(-)*-specific downregulated genes are highly enriched for GO terms in protein biosynthesis, protein degradation, unfolded protein response, protein homeostasis, development and cell division (Figure 3E; Table S4B). Thus, our results suggest that in response to *hcf-1* inactivation and *sir-2.1* overexpression, DAF-16 specifically induces longevity assurance genes to combat toxic cellular insults/stressors and extend lifespan without strongly affecting developmental, and protein homeostasis pathways.

DAF-16 directly binds a consensus DAF-16 binding element (DBE) to regulate the expression of many downstream target genes [53], [54]. To further investigate how the HCF-1/SIR-2.1-coregulated vs. the IIS-specific DAF-16 target genes might be regulated, we analyzed the 1.5 kb upstream promoter sequences of genes in each group to identify any transcription factor binding sites and regulatory elements that are overrepresented. We submitted the upstream sequences of all genes in *hcf-1/sir-2.1-*coregulated or *daf-2-*specific categories to two *de novo* motif finding algorithms, BioProspector [55] and Regulatory Sequence Analysis Tools (RSAT) [56] and focused on the top highest-scoring motifs from each algorithm. These analyses revealed four common motifs enriched in the promoters of DAF-16 targets, regardless of their responsiveness to HCF-1 & SIR-2.1 (Table S4C), suggesting that DAF-16 likely collaborates with additional yet-to-be identified co-factors in gene regulation.

We were particularly interested in the motifs that are uniquely enriched in the different groups of genes analyzed. The most notable motif highly enriched in the *hcf-1/sir-2.1*/*daf-2*-overlapping group, but not in the *daf-2*-unique group, was the DAF-16-associated element (DAE) (CTTATCA or TGATAAG), previously discovered as a sequence overrepresented in the promoters of DAF-16-regulated genes [16], [54] and shown to be directly bound by DAF-16 in *in vitro* gel shift assays [54] (Table S4C). Interestingly, the DAE represents a GATA-factor binding motif that is highly enriched in promoters of genes whose expression show age-dependent changes and whose transcription is controlled by *C. elegans* GATA-factor homologs *elt-3*, *elt-5*, and *elt-6* [57]. We further compared the expression profiles of *hcf-1(-)* and *sir-2.1*(O/E) to that of aging worms [57], and found that 23% of genes that show age-dependent changes were also represented in our *hcf-1/sir-2.1* co-regulated set (*p*-value<2.2e-16 as determined by Chi^2^ analysis). The large representation of genes that show age-dependent expression changes in the *hcf-1/sir-2.1* group correlates well with our observation that HCF-1 and SIR-2.1 together regulate aging- and stress response-specific DAF-16 downstream targets (Figure 3E). Results from the motif analysis also suggest that HCF-1 and SIR-2.1 likely engage additional transcriptional partners, such as GATA factors, in their regulation of DAF-16.

### HCF-1 forms a protein complex with SIR-2.1 and 14-3-3 proteins in *C. elegans*

**Figure 4:**
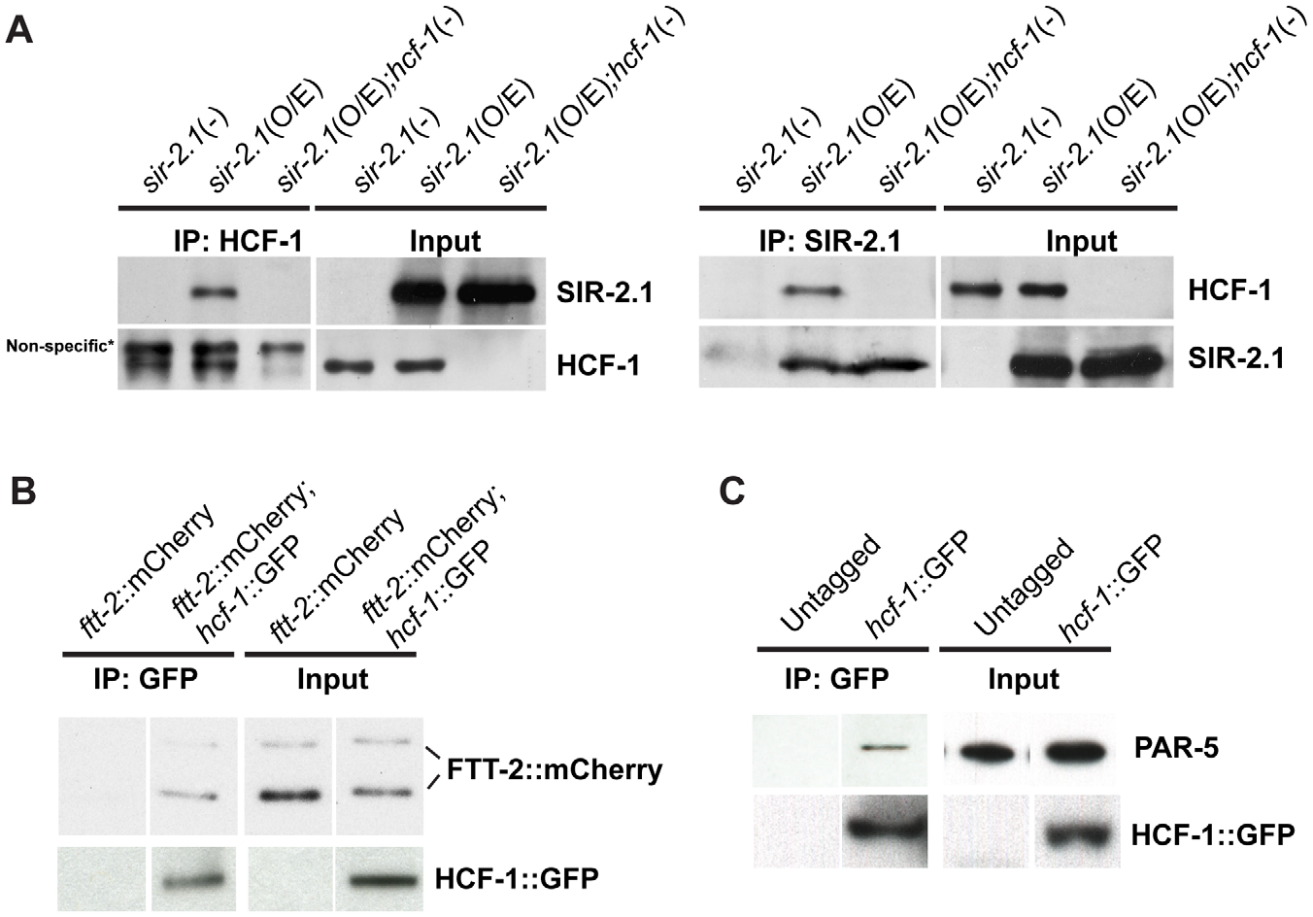
*C. elegans* HCF-1 physically interacts with SIR-2.1 and 14-3-3 proteins. (A) Lysates from *sir-2.1*(-) *(sir-2.1(ok434)), sir-2.1*(O/E) *(geIn3[sir-2.1*(O/E)*])*, and *sir-2.1*(O/E);*hcf-1(-) (hcf-1(ok559);geIn3[sir-2.1*(O/E)*])* worms were either immunoprecipitated using anti-HCF-1 antibody (left panel) or anti-SIR-2.1 antibody (right panel). The immunoprecipitated protein complexes were subsequently immunoblotted using anti-HCF-1, or anti-SIR-2.1 antibodies. (B) Lysates from *ftt-2::mCherry* or *hcf-1::GFP;ftt-2::mCherry* were immunoprecipitated with an anti-GFP antibody and blotted with anti-mCherry or anti-GFP antibodies. (C) Wild-type or HCF-1::GFP expressing worm lysates were immunoprecipitated using anti-GFP antibodies and blotted with anti-PAR-5 or anti-GFP antibody.

Our genetic and microarray analyses suggest that SIR-2.1 likely antagonizes HCF-1 to regulate DAF-16 activity. To elucidate the molecular mechanism by which SIR-2.1 may inhibit HCF-1, we first tested whether HCF-1 expression or stability is affected by SIR-2.1. We found that the mRNA and protein levels of HCF-1 did not significantly differ in strains lacking or overexpressing *sir-2.1* (data not shown). Since both SIR-2.1 and HCF-1 are known to form a protein complex with DAF-16 in *C. elegans* [18], [21], we next examined whether SIR-2.1 may also physically associate with HCF-1. We performed co-immunoprecipitation (co-IP) experiments using an affinity-purified anti-HCF-1 antibody and immunoprecipitated HCF-1 from lysates of *geIn3[sir-2.1*(O/E)*]*, worms overexpressing SIR-2.1 to a greater extent than the *pkIs1642[sir-2.1*(O/E)*]* strain we used for lifespan analysis, *hcf-1(pk924);geIn3[sir-2.1*(O/E)*]*, worms overexpressing SIR-2.1 but lacking *hcf-1*, and *sir-2-1(ok434)*, worms lacking *sir-2.1*. SIR-2.1 was co-immunoprecipitated with HCF-1 only in the *geIn3[sir-2.1*(O/E)*]* lysate (Figure 4A, left panel). A similar complex formation was also detected in reciprocal co-immunoprecipitation experiments (Figure 4A, right panel).

Since 14-3-3 proteins are proposed to bridge the physical interactions between SIR-2.1 and DAF-16, especially under stress conditions [18], [25], and our genetic data revealed that 14-3-3 likely function downstream of HCF-1 in longevity modulation, we tested a possible physical association of HCF-1 with 14-3-3 proteins. We immunoprecipitated GFP-fused HCF-1 using anti-GFP antibodies from *hcf-1::gfp;ftt-2::mCherry* or *hcf-1::gfp* strains and blotted with anti-mCherry or anti-PAR-5 antibodies to monitor mCherry-tagged FTT-2 and endogenous PAR-5 respectively. HCF-1 was able to form a protein complex with either FTT-2 or PAR-5 (Figure 4B, 4C). Consistent with the co-IP results, a search for HCF-1 binding partners using immunoprecipitation of HCF-1::GFP followed by mass spectrometrical analysis of co-purifying proteins identified the two 14-3-3 proteins FTT-2 and PAR-5 (Figure S4B). Interestingly, sequence analysis (by scansite.mit.edu) predicts that HCF-1 contains a highly significant consensus 14-3-3 binding site, suggesting HCF-1 may directly bind 14-3-3. Taken together, our data reveal that HCF-1 is a new component in the regulatory network involving SIR-2.1, 14-3-3, and DAF-16.

### Mammalian HCF-1 and HCF-2 regulate the expression of FOXO target genes

**Figure 5:**
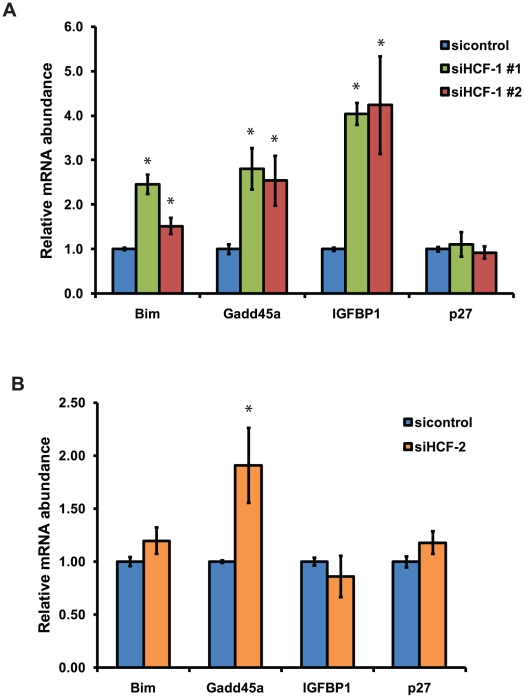
Mammalian HCF-1 and HCF-2 regulate the expression of FOXO target genes. (A) INS-1 cells treated with *HCF-1* or control siRNA. (B) INS-1 cells treated with *HCF-2* or control siRNA. mRNA levels of *Bim*, *Gadd45a*, *p27*,and *IGFBP1* were quantified using RT-qPCR and normalized to the level of *β-actin*. The mean normalized RNA level for each gene in sicontrol treated cells was set to 1. The data represented are pooled from three independent experiments and are represented as mean +/− SEM. * denotes a *p*-value<0.05 relative to sicontrol.

*C. elegans* HCF-1 belongs to a highly conserved family of proteins [38], [58], [59]. In mammals, two homologs of HCF-1 are present: HCF-1 and HCF-2 [32], [60]. Mammalian HCF-1 plays a role in transcriptional regulation and cell cycle progression, whereas the functions of HCF-2 remain unknown. SIRT1, the mammalian homolog of SIR-2.1, is known to interact with and deacetylate the DAF-16 homologs FOXO1, FOXO3, and FOXO4 and in doing so affects FOXO transcriptional activity [28], [30]. Given that HCF-1, DAF-16 and SIR-2.1 are highly conserved between *C. elegans* and mammals, we tested whether mammalian homologs of HCF-1 could affect the transcription of FOXO- and SIRT1- co-regulated target genes. Since mammalian HCF-1 is required for proper cell cycle progression, we employed a transient knockdown approach by transfecting siRNA duplexes targeting the *HCF-1* gene into INS-1 rat insulinoma cells. We used two different *HCF-1* siRNA duplexes to control for specificity, and found that *HCF-1* knockdown did not substantially affect the expression of *HCF-2* mRNA as assessed by reverse transcription-quantitative PCR (RT-qPCR) (Figure S5). We examined the expression of *Bim*, *a* proapoptotic factor, *Gadd45a*, which is involved in DNA damage repair, *IGFBP1*, an insulin-like growth factor-binding protein, and *p27*, a cyclin-dependent kinase inhibitor. These represent FOXO target genes which are affected by SIRT1 deacetylation of FOXO [28], [30]. Depletion of *HCF-1* resulted in a significant increase in the levels of *Bim*, *Gadd45a*, and *IGFBP1* transcripts, but did not affect *p27* expression (Figure 5A). Consistent results were obtained with the two different *HCF-1*-targeting siRNA duplexes. We next tested whether HCF-2 could also affect FOXO target gene expression. Similar to *HCF-1* knockdown, cells treated with *HCF-2* siRNA exhibited increased expression of *Gadd45a* and no change in *p27*. However, unlike the case with HCF-1, cells depleted of *HCF-2* did not show any significant changes in *Bim*, or *IGFBP1* transcripts (Figure 5B). Our data reveal that HCF proteins negatively regulate the expression of a subset of FOXO and SIRT1 transcriptional target genes. Furthermore, HCF-1 appears to play a more substantial role in regulating FOXO target genes relative to HCF-2. The observation that HCF-1 and HCF-2 have specific effects on a subset of FOXO targets tested is also consistent with our findings in *C. elegans* suggesting HCF-1 to be a specificity factor for DAF-16/FOXO.

### Mammalian HCF-1 and HCF-2 physically associate with FOXO3 and SIRT1

**Figure 6:**
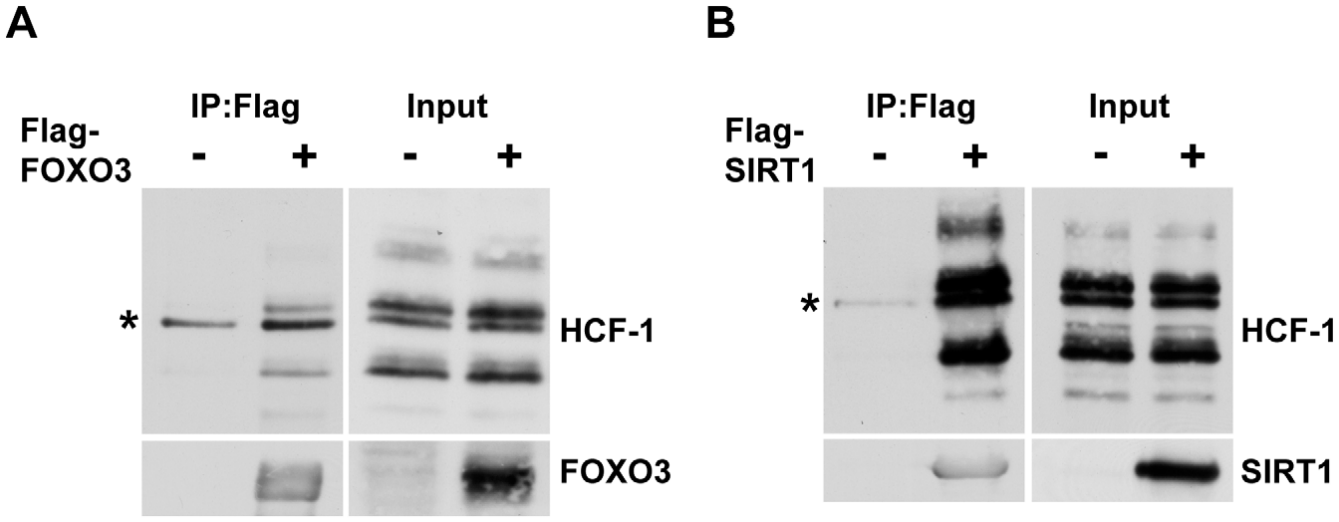
Mammalian HCF-1 physically associates with FOXO3 and SIRT1. (A, B) HEK293T cells were transfected with plasmids encoding Flag-FOXO3 (A) or Flag-SIRT1 (B). Cell lysates were collected 48 hours later and incubated with anti-Flag-conjugated agarose beads. Immunoprecipitated protein complexes were analyzed by western blot using anti-HCF-1, anti-FOXO3 or anti-SIRT1 antibodies. HCF-1 is known to be proteolytically processed and is detected as multiple bands on SDS-PAGE [76]. * denotes a non-specific band.

In *C. elegans*, HCF-1 is able to physically associate with both DAF-16 and SIR-2.1 (Figure 4A and [21]). We therefore hypothesized that mammalian HCF proteins will also participate in protein complexes with FOXO3 and SIRT1. To examine the physical interactions between these proteins, we transfected HEK293T cells with plasmids encoding either Flag-tagged FOXO3 or Flag-tagged SIRT1. We then performed co-immunoprecipitation experiments with these cell lysates by using Flag-antibody conjugated agarose beads. Both FOXO3 and SIRT1 were found to interact with the endogenous mammalian HCF-1 protein (Figure 6A, 6B; Figure S6A). We also tested whether the closely related HCF-2 protein could also physically interact with FOXO3 and SIRT1. Since antibodies capable of detecting endogenous HCF-2 are not available, we performed co-immunoprecipitation experiments using overexpressed Flag-FOXO3, Flag-SIRT1, and HA-tagged HCF-2. We found that HCF-2 was also present in a protein complex with FOXO3 and SIRT1 when overexpressed (Figure S6B), similar to HCF-1. These results indicate that the physical associations between HCF-1, DAF-16 and SIR-2.1 are highly conserved between *C. elegans* and mammals.

## Discussion

**Figure 7:**
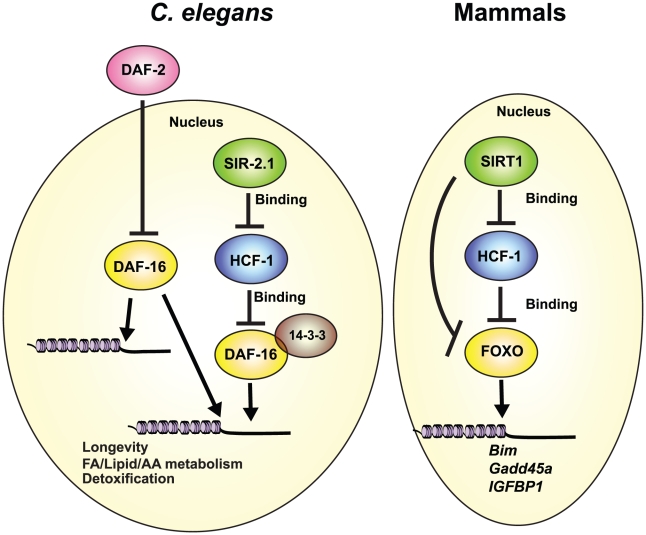
Conserved regulation of DAF-16/FOXO by HCF-1 and SIR-2.1/SIRT1. We propose that *C. elegans* HCF-1 and SIR-2.1 coordinate to fine-tune the transcriptional activity of DAF-16 on a distinct subset of potential target genes. DAF-16 target genes responsive to the *hcf-1/sir-2.1* pathway largely overlap with a small subset of IIS-regulated genes, and are specialized in longevity determination, cellular defense, and lipid/fatty acid/amino acid homeostasis. HCF-1 likely represses DAF-16 by forming a complex with SIR-2.1 and 14-3-3, and antagonizing their abilities to stimulate DAF-16. This functional relationship is highly conserved as mammalian HCF proteins also repress the expression of multiple FOXO/SIRT1 target genes and reside in protein complexes with SIRT1 and FOXO3. Our results highlight HCF proteins to be key components of the regulatory network linking SIR-2.1/SIRT1 and DAF-16/FOXO in diverse organisms.

The highly conserved FOXO transcription factors are master regulators of diverse biological processes [61] and as such, their transcriptional activities are tightly controlled [18]–[23]. Although a number of different transcriptional co-factors of DAF-16/FOXO have been identified, little is known about how they functionally interact to fine-tune DAF-16/FOXO activity, and in particular, how they may collaborate to affect DAF-16-mediated lifespan extension. In this study, we identified the DAF-16 nuclear co-repressor HCF-1 as an integral component of the regulatory network involving SIR-2.1/SIRT1, 14-3-3, and DAF-16/FOXO with major consequences to both organismal aging and stress response. Our data indicate that in *C. elegans*, HCF-1 likely functions downstream of SIR-2.1, and upstream of 14-3-3, to regulate a distinct subset of DAF-16 target genes to affect longevity and oxidative stress response. This regulatory pathway is highly conserved, as mammalian HCF proteins also impact the expression of SIRT1/FOXO co-regulated transcriptional targets, and HCF proteins participate in protein complex formation with SIR-2.1/SIRT1, 14-3-3, and DAF-16/FOXO in worms and in mammals (Figure 7).

Our expression profiling studies indicate that the set of DAF-16 target genes co-regulated by *sir-2.1*, *hcf-1*, and *daf-2* (area “a” of Figure 3D) is enriched for previously identified longevity-associated genes (annotated as “aging” in GO), whereas the IIS-specific targets (area “g” of Figure 3D) are not. This is somewhat unexpected as the *hcf-1* mutant and *sir-2.1* overexpressor strains exhibit lifespan extension phenotypes that are much milder than that of the *daf-2* mutant. Interestingly, this correlates well with the degree of expression change observed for many of the shared DAF-16 target genes, as they often exhibit more robust expression changes in the *daf-2(-)* profile compared to the *sir-2.1*(O/E) or *hcf-1(-)* profiles. An implication from this observation is that the co-regulated gene set is particularly important for longevity determination, and may thus contain additional targets important for prolonged lifespan that are not currently known to affect aging.

Our previous genetic findings indicated that reduced insulin signaling synergizes with inactivation of *hcf-1* to affect longevity and DAF-16-mediated gene regulation [21]. We interpreted those results to suggest that IIS and *hcf-1* likely act independently to regulate DAF-16/FOXO. However, a caveat of that interpretation is that the *daf-2* mutant we examined was not a null mutant, and formally, loss of *hcf-1* can further decrease IIS signaling to further increase lifespan. Similarly, the genetic relationship between the insulin signaling pathway and *sir-2.1* has been unclear due to several conflicting reports [18], [24]. In the current study, a comparison of the DAF-16-regulated gene expression changes in response to either *daf-2* mutation, *hcf-1* inactivation, or *sir-2.1* overexpression indicates that a large majority of the HCF-1/SIR-2.1 co-regulated DAF-16 target genes are similarly regulated by reduced IIS. It is possible that upon downregulation of IIS, the majority of DAF-16 migrates into the nucleus but is still subject to regulation by nuclear co-factors. Under this scenario, SIR-2.1 and HCF-1 may be acting as additional “gate keepers” to control DAF-16 activation in the face of reduced IIS. In addition, we saw that the insulin/IGF-1-like peptide, *ins-7*, which was shown to act as a *daf-2* agonist [16], was significantly repressed by *hcf-1* inactivation and *sir-2.1* overexpression (Table S3). Thus, a possible feedback mechanism in which *hcf-1* inactivation or *sir-2.1* activation leads to further inhibition of IIS may also explain the genetic results observed with reduced IIS and *hcf-1* inactivation or *sir-2.1* overexpression.

Our motif analyses revealed additional factors that are likely involved in the regulation of DAF-16 by HCF-1 and SIR-2.1 in *C. elegans*, in particular the aging-related GATA-factor homologs (ELT-3, -5, -6) known to bind the DAE element, a consensus motif enriched in many of the HCF-1/SIR-2.1 co-regulated genes [57]. Of note, the DAE sequence also shares close resemblance to the mammalian transcription factor Evi1 binding site. Although the *C. elegans* Evi1 homolog, *egl-43*, has been shown to be involved in early development [62], a function in longevity and stress response has not been reported. Future functional analysis of HCF-1/SIR-2.1 and ELT-3, -5, -6, and EGL-43 will likely yield new insights into additional layers of DAF-16 regulation.

Considering the high conservation of DAF-16/FOXO-related pathways, it is not surprising that the regulatory relationship among HCF-1, SIR-2.1 and DAF-16 we uncovered in worms turns out to be conserved in mammals. Our findings in mammalian cells are nevertheless very exciting as they implicate the HCF proteins to be key components linking FOXO and SIRT1, two critical master regulators of physiology in mammals. Our results indicate that while both mammalian HCF-1 and HCF-2 are able to interact with SIRT1 and FOXO3, HCF-1 has a greater effect on FOXO target gene expression. Interestingly, while both mammalian HCF-1 and HCF-2 as well as *C. elegans* HCF-1 are able to support the formation of the Herpes Simplex Virus VP16-transcriptional complex, only mammalian and *C. elegans* HCF-1 are able to promote VP16 transcriptional activity [32]. Thus, it appears that the evolutionarily conserved functions of HCF proteins are retained in mammalian HCF-1. Alternatively, HCF-1 and HCF-2 likely have tissue-specific functions and are regulated differently under different cellular contexts.

While our data indicate that parallel regulatory mechanisms are shared between *C. elegans* and mammalian HCF-1, they also suggest the modes of regulation between HCF-1, SIRT1, and FOXO in mammals are likely more complex than what is observed in *C. elegans*. We note that in the case of the mammalian FOXO target genes *Bim* and *IGFBP1*, HCF-1 and SIRT1 appear to affect FOXO target gene expression in a similar manner (Figure 5A and [28], [30]), and thus would appear to act in concert rather than antagonistically. On the other hand, HCF-1 and SIRT1 appear to have antagonistic effects on the FOXO target gene *Gadd45a*. It is important to keep in mind that in mammals, SIRT1 regulation of FOXO transcription factors is complex; in some instances SIRT1 acts as a repressor and in other cases as an activator of FOXO [28], [30], while in *C. elegans* the predominant role of SIR-2.1 is as an activator of DAF-16. It is likely that in mammals, the interplay between SIRT1 and HCF-1 results in collaborative as well as antagonistic effects on FOXO transcriptional activity in a gene- and context-dependent manner. Future genome-wide studies examining the effects of HCF-1 on FOXO/SIRT1-regulated gene expression will provide further insights into the relationship between HCF-1 and SIRT1.

We found that HCF-1 physically associates with DAF-16/FOXO and SIR-2.1/SIRT1 in both worms and mammals. Previous studies in *C. elegans* indicate that 14-3-3 proteins act as bridging molecules that bring SIR-2.1 and DAF-16 into a protein complex in the nucleus [18], [25]. Interestingly, our data suggest 14-3-3 proteins also physically associate with HCF-1. This raises the question of how these different molecules coordinately interact to affect each other's activities. An intriguing model may be that HCF-1 normally binds 14-3-3/DAF-16 and dampens the ability of DAF-16 to activate its target genes; upon appropriate upstream signals, SIR-2.1 ejects HCF-1 off the complex and induces full activation of DAF-16. Whether 14-3-3 proteins are also involved in the regulation of FOXO by SIRT1 and HCF in mammals remain to be investigated. In addition, SIRT1 is known to regulate FOXO transcriptional activity by directly deacetylating FOXO proteins and the FOXO co-activator PGC1α in mammals [63]–[65]. SIRT1 may affect multiple FOXO responses by deacetylating FOXO and specific FOXO co-regulators to achieve activation and/or repression of the appropriate target genes. Future investigation into whether SIRT1 also regulates HCF-1 via deacetylation and whether deacetylation will disrupt protein complexes involving SIRT1/HCF-1/FOXO will provide new insights into the functional interactions among these key longevity determinants.

In conclusion, our findings establish a novel link between two evolutionarily conserved DAF-16/FOXO regulators. This study expands our understanding of the complex role that nuclear factors play in determining the specificity of DAF-16/FOXO activity. These results further implicate HCF-1 as a novel factor that may affect mammalian aging and age-related pathologies through interactions with SIRT1 and FOXO.

## Materials and Methods

### *C. elegans* strains

All strain stocks were kept at 16°C and grown under standard growth conditions [66]. The strains used are: Wild type N2, *hcf-1(pk924)*, *daf-16(mgDf47);hcf-1(pk924)* [21], IU372.1 *sir-2.1(ok434)* (7 times outcrossed in our lab), NL3908 *pkIs1641* [*unc-119*], NL3909 *pkIs1642 [unc-119 sir-2.1]* [18], IU91.1 *pkIs1641 [unc-119]* (1X outcrossed in our lab), IU94 *pkIs1642 [unc-119 sir-2.1]*(1X outcrossed in our lab), *geIn3[sir-2.1 rol-6(su1006)]* [24] (1X outcrossed in our lab), *ftt-2(n4426)* [18] (3X outcrossed in our lab), *rwIs23* [*hcf-1(pk924);Phcf-1::GFP unc-119]*, GR1680 *rwIs23[Phcf-1::GFP; unc-119]; IsB[pCR270(Pftt-2::ftt-2:: Spep-TEV-mCherry::ftt-2-3′UTR; Cb_unc-119)]*, *rwIs9[Phcf-1::hcf-1::GFP Pmec-7::RFP]*. Standard genetic methods were utilized to construct the following strains: *sir-2.1(ok434) hcf-1(pk924)*, *hcf-1(pk924);pkIs1642[sir-2.1*(O/E)*], hcf-1(ok559);geIn3[sir-2.1 rol-6(su1006)], ftt-2(n4426);hcf-1(pk924)*. *daf-16(mgDf50); pkIs1642[sir-2.1*(O/E)*]* was a gift from M. Viswanathan and L. Guarente at MIT [43].

### Lifespan analysis

All lifespan assays were performed at 25°C, unless otherwise noted, on Nematode Growth Media (NGM) plates seeded with *E. coli* OP50 or RNAi bacteria. For experiments using OP50, bacteria was grown overnight at 37°C, OD measured after growth and concentrated to OD 7.5 (5X OP50) or used directly, at OD 1.5 (1X). 35 mm NGM plates were seeded with 150 uL of OP50 for egglay plates and dried at room temperature. Plates that would be used for transferring worms throughout the lifespan assay were prepared by adding FUDR to OP50 culture to a final concentration of 50 ug/mL per plate, seeding 150 uL/plate, drying at room temperature, and storing at 4°C until use. For RNAi experiments, HT115 bacteria containing vectors expressing dsRNA were grown at 37°C in LB with 100 ug/mL carbenicillin and 15 ug/mL tetracycline to OD 0.8, induced with 4 mM IPTG for 4 hrs at 37°C, and either concentrated to OD 7.5 and seeded, or seeded at OD 1.5 (1X). RNAi plates were also induced with 4 mM IPTG before use. Well-fed gravid adult worms were allowed to lay eggs at room temperature and the progeny were grown at 25°C until young adult/early gravid adult stage. The synchronized adults were transferred to fresh FUDR-containing plates at Day 0, 2, and 4 of adulthood. For lifespan assays carried out at 20°C, worms were incubated at 25°C for the first three days of adulthood to reduce vulva protrusion defects. The adult worms were scored every other day and worms that did not move when gently prodded by a platinum wire pick were recorded as dead. Worms that bagged, crawled onto the wall of the plate, or had a large protruding vulva were censored on the day of the event. All survival data were analyzed using Kaplan-Meier statistics (SPSS software) to generate statistical values and survival curves. *p*-values were calculated using the log-rank test. Kaplan-Meier log rank test was employed to determine whether independent experiments displayed statistically similar trends using a cutoff of *p*-value>0.05. Based on these criteria, data from independent experiments were pooled whenever possible to increase statistical power.

### Stress assays

#### Paraquat

50–60 synchronized worms were grown on three 60 mm NGM/OP50 plates (per strain) at 25°C until day two of adulthood, either directly transferred or washed off the plates with M9 buffer and dispensed into three wells of a 24-well culture plate, and paraquat (Sigma) added to 150 mM or 200 mM final concentration. Plates were kept covered by aluminum foil to prevent excessive light from degrading paraquat, and rocked on a shaker at 25°C. Survival was scored at the indicated time points after paraquat exposure.

#### *tert*-Butyl hydroperoxide

Synchronized worms were grown on OP50 plates until day one of adulthood and transferred onto plates containing 6 mM *tert*-Butyl hydroperoxide (*t*-BOOH) (Sigma). Survival was scored at indicated time points after treatment.

Kaplan Meier analysis and Log-rank statistics (SPSS software) were used to generate survival curves, calculate mean survival, and compute statistics. Log-rank test was also employed to determine whether independent experiments displayed statistically similar trends using a cutoff of *p*-value>0.05. Based on these criteria, data from independent experiments were pooled whenever possible to increase statistical power. The mean variation in survival of each strain as compared to either wild-type or *pkIs1641[sir-2.1*(wt)*]* was calculated and further analyzed by Linear Mixed model analysis [67] to obtain averaged mean variations relative to control from two or three independent experiments. *hcf-1(-)* and *sir-2.1(-) hcf-1(-)* or *sir-2.1*(O/E) and *hcf-1(-)*;*sir-2.1*(O/E) were entered as “fixed effect” and experiments as “random effect”. Linear Mixed model analysis allows statistical evaluation of differences between various treatments (mutants) by taking into account the experimental variation.

### RNA isolation and microarray preparation

For *hcf-1(-)* microarrays, total RNA was purified from synchronized L4 or young adult(YA) worms. Worms were synchronized by allowing hypochlorite-treated eggs to hatch in M9 buffer for 20 hrs at 16°C, and plating 500 L1 stage worms onto each of 5–6 10 mm NGM plates seeded with 3X OP50 bacteria. 6 biological replicates of *hcf-1(-)/daf-16(-);hcf-1(-)*, two replicates of *hcf-1(-)*/N2 were prepared. The synchronized populations were grown to L4 or YA stage at 25°C and harvested by washing off the plates with M9 buffer and freezing the worm pellet in liquid nitrogen. Total RNA was isolated using Tri-reagent (Molecular Research Center, Inc.) [68] and purified with the RNeasy kit (Qiagen). cRNA synthesis/amplification, Cy3/Cy5 dye labeling, and hybridization onto Agilent 4X44K *C. elegans* oligonucleotide microarrays were performed as previously described [49]. Half the arrays were dye-flip replicates in each comparison.

Details on *sir-2.1*(O/E) microarrays will be published elsewhere (Rogers*, Jan*, Ashraf, and Murphy, in preparation). *daf-2(-)* microarray data were published in [49].

### Microarray analysis

#### *hcf-1(-)* microarrays

Hybridized microarray slides were washed according to Agilent instructions , and images were scanned using an Axon Instruments GenePix 4000B scanner, reading at wavelengths of 635 nm and 532 nm (Axon Instruments, http://www.axon.com) [69]. The arrays were scanned at three different PMT settings to capture spots with low and high signal, and later combined to create a single dataset. The image data were uploaded onto the Princeton University MicroArray database (PUMA [http://puma.princeton.edu]). Log_2_ transformed fold change data were acquired after normalizing, filtering for array and spot quality, collapsing replicate spots to a mean value on PUMA.

Data for *sir-2.1*(O/E) and *daf-2(-)* arrays were similarly normalized and processed on PUMA.

#### SAM analysis

Log_2_ transformed fold change data with no cutoff were submitted to SAM [48]. One class analysis was used to identify genes significantly and consistently changed in each database. Two-class unpaired analysis was used to identify genes similarly and divergently changed between different datasets. Genes found to be significantly changed at 0% FDR in only *hcf-1(-)*, *sir-2.1*(O/E), or *daf-2(-)* using one-class analysis, and similarly and divergently changed between different datasets using two-class unpaired analysis were combined and sorted based on the SAM output to generate heat maps using Treeview [70].

#### Gene Ontology classification

Worm Base IDs (WBID) of genes identified in SAM were pasted into the Functional annotation clustering tool in DAVID (http://david.abcc.ncifcrf.gov/) for gene annotation enrichment analysis [50], [71]. Functional annotation clustering was performed with the default criteria and enrichment score for each annotation cluster was determined.

#### Upstream regulatory motif analysis

1.5 kb upstream sequences were submitted to BioProspector (http://ai.stanford.edu/~xsliu/BioProspector/) [55] and RSAT (http://rsat.ulb.ac.be/rsat/) [56] to identify overrepresented cis-regulatory elements. An oligonucleotide length of 8 bp was specified for both algorithms. The highest scoring (most significantly enriched) 10 motifs from BioProspector and 5 motifs from RSAT were obtained. As BioProspector returns the same sequences multiple times, only unique motifs were reported. Motifs were displayed in WebLogo (weblogo.berkeley.edu) [72]. The matrices associated with each motif were submitted to the TomTom motif comparison tool (http://meme.sdsc.edu/meme/cgi-bin/tomtom.cgi) [73] to compare against a database of known transcription factor binding sites (Transfac).

### Immunoprecipitation and mass spectrometry (*C. elegans*)

Immunoprecipitation was performed as described [21]. For HCF-1/SIR-2.1 co-IPs, mixed stage worms were grown on plates, harvested, and sonicated in IP lysis buffer (50 mM HEPES pH 7.5, 1 mM EDTA, 150 mM NaCl, 10% Glycerol, 0.1% Triton X-100, 1 mM sodium fluoride, 2.5 mM sodium orthovanadate, 1 mM PMSF, and Complete (EDTA-free) protease inhibitor cocktail) and lysates cleared by centrifugation. Lysates were incubated with either affinity purified guinea-pig anti-HCF-1 antibody [21] or rabbit anti-SIR-2.1 antibody (Novus Biologicals) at 4°C overnight. Immunocomplexes were incubated with trysacryl protein A-agarose beads (Pierce) at 4°C for four hours, washed four times with IP lysis buffer, and eluted by boiling in SDS sample buffer. Eluted protein complexes were analyzed by western blotting using the anti-HCF-1, anti-SIR-2.1, or anti-actin (Chemicon, clone C4) antibodies.

For mass spectrometry and 14-3-3 co-IPs, GFP-tagged HCF-1 was purified from mixed stage *C. elegans*, using a previously reported method [74] with slight modifications. In short, worms were grown in liquid culture as mixed stages to a density of 4000 worms/mL. Worms were washed into lysis buffer (50 mM HEPES at pH 7.4, 1 mM EGTA, 1 mM MgCl2, 150 mM KCl, 10% (v/v) glycerol, protease and phosphatase inhibitors), drop-frozen in liquid nitrogen, and ground using a mortar and pestle. Resulting powder was thawed and NP-40 was added to 0.05% (v/v). Immunoprecipitations were conducted on a 20,000 g supernatant of this extract, using monoclonal mouse-anti-GFP antibody (Invitrogen) coupled to Protein A resin (Biorad). Immunoprecipitated proteins were eluted using 100 mM glycine at pH 2.6. For co-IPs, eluted protein complexes were analyzed by western blotting using anti-mCherry (Ruvkun Lab, MGH Boston) or rabbit anti-PAR-5 (a kind gift from K.J. Kemphues, Cornell University) antibodies. For mass-spectrometrical analysis, immunoprecipitated proteins were eluted using 100 mM glycine at pH 2.6. Eluted proteins were visualized by silver-stained SDS-PAGE and identified by mass spectrometry. For the latter, samples were digested using trypsin and the resulting peptides were separated via nano-capillary liquid chromatography and identified by online tandem mass spectrometry (LTQ-XL, Thermo). Mass spectra were searched against the current wormpep database using Sequest (Thermo) and DTASelect [75].

As a negative-control for the mass-spectrometrical analysis, an identical purification was conducted using *C. elegans* expressing only untagged endogenous HCF-1. IP and negative-control were compared using Contrast [75].

### Plasmids, shRNA, and siRNA for mammalian cells

Flag-FOXO3 and Flag-SIRT1 were obtained from Addgene and have been described previously [28]. The plasmids encoding HA-HCF-1 and HA-HCF-2 were generated by cloning the human HCF-1 and HCF-2 cDNA into the vector pCMV-HA (Clontech). The plasmid encoding the short-hairpin RNA targeting the human *SIRT1* gene was generously provided by W.L. Kraus [8]. The plasmid encoding shRNA targeting the firefly *luciferase* gene was generously provided by L. Qi (Cornell University). siRNA duplexes directed against rat *HCF-1* and *HCF-2* were purchased from Dharmacon and targeted the following sequences: siHCF-1 #1: 5′-GGAAGAGACTGAAGGCAAA-3′; siHCF-1 #2: 5′-AGAACAACATTCCGAGGTA-3′; siHCF-2: 5′- GGGAATGGTTGAATATGGA-3′. Non-targeting control siRNA was also from Dharmacon. Cells were collected 48 hours post-transfection, or treated for an additional 6 hours with nicotinamide (10 mM, Sigma).

### Cell culture and transfection

HEK293T were maintained in DMEM containing 4.5 g/L glucose and 10% calf serum and were transfected with the indicated plasmids using calcium phosphate. INS-1 cells were maintained in RPMI-1640 medium containing 11.1 mM glucose, 10% fetal bovine serum, 1 mM pyruvate, 10 mM HEPES, and 50 µM 2-mercaptoethanol. INS-1 cells were transfected with siRNA at a concentration of 10 nM using Lipofectamine RNAiMax (Invitrogen). siRNA transfections were performed twice, 24 hours apart, and cells were collected 24 hours after the second transfection.

### Reverse-transcription coupled quantitative PCR (RT-qPCR)

RNA was isolated from mammalian cells using Trizol reagent and was reverse-transcribed using Superscript III First-Strand kit (Invitrogen). cDNAs were analyzed by quantitative-PCR using the SYBR Green system on a Roche LightCycler 480 real time PCR machine and quantified relative to a standard curve. *β-actin* was used as an internal control. The following primers were used: *β-actin* forward: 5′- CTAAGGCCAACCGTGAAAAG-3′; : *β-actin* reverse: 5′-AACACAGCCTGGATGGCTAC-3′; *HCF-1* forward: 5′-GCTGGAAAAGCTCCTGTCAC-3′; *HCF-1* reverse: 5′- CACTCATCTGTGGGTTGCTG-3′; *HCF-2* forward: 5′- TTGAAAGCAGAGCAATGGTG-3′; *HCF-2* reverse: 5′- AGTCGGGTACGTCTGCATTT-3′; *Bim* forward: 5′- GCCCCTACCTCCCTACAGAC-3′; *Bim* reverse: 5′- CAGGTTCCTCCTGAGACTGC-3′; *p27* forward: 5′- GTGGACCAAATGCCTGACTC-3′; *p27* reverse: 5′- TTCTGTTCTGTTGGCCCTTT-3′; *Gadd45a* forward: 5′- GCAGAGCTGTTGCTACTGGA-3′; *Gadd45a* reverse: 5′- TGTGATGAATGTGGGTTCGT-3′; *IGFBP1* forward: 5′- CTGCCAAACTGCAACAAGAA-3′; *IGFBP1* reverse: 5′- TTCCCACTCCATGGGTAGAC-3′.

### Immunoblotting and immunoprecipitations (mammalian cell culture)

For co-immunoprecipitation experiments, HEK293T cells were transfected with the indicated plasmids. 48 hours after transfection, cells were lysed in lysis buffer (50 mM Tris-HCl pH 8.0, 100 mM NaCl, 2 mM EDTA, 1% TritonX-100, 10 mM NaF, 1 mM sodium orthovanadate, 1 mM PMSF, 10 mM nicotinamide, 1 mM trichostatin A, and Roche complete protease inhibitor cocktail). Cell extracts were incubated with either Flag- or HA-conjugated agarose beads (Sigma) overnight at 4°C. Beads were washed 5 times in lysis buffer and eluted by boiling in SDS sample buffer. Immunoprecipitates were analyzed by western blotting using the following antibodies: anti-HA (Covance), anti-FOXO3 (Upstate), anti-SIRT1 (gift from W.L. Kraus), anti-HCF-1 (Bethyl Labs).

## Supporting Information

Figure S1 *hcf-1* is epistatic to *sir-2.1* in oxidative stress response. (A–D) The average mean variation in survival of each strain relative to wild-type N2 or *pkIs1641* is displayed. Mean survival is calculated starting from the time of *t*-BOOH (A–B) or paraquat (C–D) exposure. The data represent pooled data from two independent experiments. * denotes a *p*-value<0.05 compared to *sir-2.1(-) hcf-1(-)*. All stress experiments were carried out at 25°C. Quantitative data and statistical analyses are displayed in Table S1C–S1F.
(EPS)

Figure S2 Low copy overexpression of *sir-2.1* extends lifespan. (A) Lifespans of wt, *sir-2.1*(wt) (*pkIs1641), sir-2.1*(O/E) *(pkIs1642)*, *sir-2.*1(wt)-1X (one time outcrossed *pkIs1641*, strain IU91.1), *sir-2.1*(O/E)-1X (one-time outcrossed *pkIs1642*, strain IU94) are displayed. We found that the *sir-2.1* overexpressor strain continues to extend lifespan after outcrossing into our lab N2 strain. See Table S1G for quantitative and statistical data. (B) The lifespan extension by *pkIs1642 [sir-2.1*(O/E)*]* is suppressed by *sir-2.1* knockdown as previously reported [18]. Lifespans of Strain+RNAi combinations are displayed. To ensure significant knockdown of SIR-2.1, worms were exposed to RNAi for three generations before proceeding with the experiment. See also Table S1H. Lifespans were carried out at 25°C. (C) A subpopulation of RNAi-treated worms used in (B) were lysed and analyzed by western blotting to measure SIR-2.1 protein levels in order to confirm efficient knockdown. SIR-2.1 levels are substantially reduced in *sir-2.1* RNAi treated strains. (D) mRNA levels of *sir-2.1* are quantified by RT-qPCR and normalized to *act-1*. Similar to protein levels, mRNA levels of *sir-2.1* are significantly diminished upon RNAi treatment.
(EPS)

Figure S3 *14-3-3* knockdown suppresses lifespan increase by *hcf-1(pk924)* mutation and *sir-2.1* overexpression but not by *daf-2(e1370)* mutation. (A–C) Worms of indicated genotypes were grown on vector (control) RNAi bacteria at 16°C until young adulthood and subsequently transferred to either control or *ftt-2* (Ahringer) RNAi at 25°C for the remainder of the experiment. Pooled data from two independent experiments are shown. See Table S2C for quantitative data. (D) Worms of indicated genotypes were grown on OP50 bacteria at 25°C throughout the experiment and pooled data from two independent experiments are shown (Also see Table S2D.)
(EPS)

Figure S4 HCF-1 physically interacts with FTT-2 and PAR-5. (A) *hcf-1(pk924)* worms were grown on plates containing vector control, non-specific (*ftt-2* and *par-5*) or gene-specific (*ftt-2gs* or *par-5gs*) *14-3-3* RNAi until young adult stage and protein levels analyzed by western blotting using anti-FTT-2 or anti-PAR-5 antibodies. Actin was used as a loading control. (B) Sequences of the peptides from FTT-2 and PAR-5 proteins, which were identified in the mass spectrometrical analysis of HCF-1::GFP-bound proteins, are listed. *represents peptides that are common to both FTT-2 and PAR-5.
(TIF)

Figure S5 Specific knockdown of *HCF-1* and *HCF-2* by siRNA. INS-1 cells transfected with *HCF-1* (A) or *HCF-2* siRNA (B) were analyzed by RT-qPCR and Western blotting. (A) Two different *HCF-1* targeting siRNA produced similar effects on FOXO target gene expression. Cells transfected with siHCF-1 #1 exhibited a moderate increase in *HCF-2* expression. *HCF-2* was not affected by siHCF-1 #2. (B) *HCF-1* siRNA substantially reduced HCF-1 protein levels. ** indicates a non-specific band. (C) Knockdown of *HCF-2* did not affect *HCF-1* expression. Values are normalized to the level of *β-actin*. The mean normalized mRNA level for each gene in sicontrol treated cells was set to 1. The data represented are pooled from three independent experiments and are represented as mean +/− SEM. * denotes a *p*-value<0.05.
(TIF)

Figure S6 Mammalian HCF homologs physically interact with FOXO3 and SIRT1. HEK293T cells were co-transfected with plasmids encoding HA-HCF1 (A) or HA-HCF2 (B) and either Flag-FOXO3 or Flag-SIRT1. Cell lysates were collected 48 hours later and incubated with either anti-Flag- or anti-HA-conjugated agarose beads. Immunoprecipitated protein complexes were analyzed by western blotting using the indicated antibodies. HCF-1 is known to be proteolytically processed and is detected as multiple bands on SDS-PAGE.
(TIF)

Table S1 Lifespan and oxidative stress phenotypes of *hcf-1* and *sir-2.1* strains. All survival analyses were done using SPSS software using Kaplan Meier analysis and log-rank test to compute *p*-values. *p*-value<0.05 is considered statistically significant. (A) Experiments #1 and #2 were conducted using a lower concentration of bacteria (1X, see Materials and Methods). Experiments #3 and #4 were done on 5X OP50 bacteria. Although the *sir-2.1(ok434)* mutants have been previously reported to exhibit a slightly shorter lifespan than that of wild-type worms, we observed variable results where the *sir-2.1(ok434)* mutants tended to live shorter under assaying conditions with lower food (#1&2) and longer on more concentrated bacteria lawns (#3&4). However, whether different bacteria food concentration is the cause of the variability of the *sir-2.1* mutant lifespan needs further investigation in the future. Nevertheless, we found that all four independent lines of the double mutants exhibited lifespans similar to that of *hcf-1(pk924)* single mutant worms, and significantly longer than that of *sir-2.1(ok434)* mutants. *sir-2.1(-) hcf-1(-)* (#1–4) represent independent isolates obtained from a cross. * Shown in Figure 1A. Data for each strain, except for *sir-2.1*(-) *hcf-1*(-) (#2–4), are pooled from four independent experiments. Data for *sir-2.1*(-) *hcf-1*(-) (#2–4) double mutant lines are pooled from two experiments. LS = Lifespan. (B) Experiments were conducted using 5X concentrated OP50 bacteria. *hcf-1(pk924);pkIs1642* (#1–5) represent independent isolates obtained from a cross. * Shown in Figure 1B. Data for each strain are pooled from three independent experiments. (C) Survival of worms treated with 6 mM *t*-BOOH was monitored. Linear Mixed model analysis was used to calculate the averaged percent variation relative to wt. Linear Mixed model analysis allows statistical evaluation of differences between various treatments (mutants) by taking into account the experimental variation. *p*-value<0.05 is considered significantly different from control. For Figure 1C, data from two independent experiments as well as two genotypically identical *sir-2.1(-) hcf-1(-)* double mutants were pooled and analyzed using Kaplan Meier and log-rank statistics. (D) Survival of worms treated with 6 mM *t*-BOOH was monitored. We used Linear Mixed model analysis to calculate the averaged percent variation relative to wt or *pkIs1641*. For Figure 1D, data from two genotypically identical *hcf-1(-);pkIs1642[sir-2.1*(O/E)*]* strains were pooled. (E) Survival of worms treated with 150 mM paraquat in M9 buffer was monitored. For Figure 1E, data from two independent experiments as well as four genotypically identical *sir-2.1(-) hcf-1(-)* double mutants were pooled. (F) Survival of worms treated with 200 mM paraquat in M9 buffer was monitored. Data from two genotypically identical *hcf-1(-);pkIs1642[sir-2.1*(O/E)*]* strains were pooled and displayed in Figure 1F. (G) Graph shown in Figure S2A. *sir-2.1*(wt)-1X (one-time outcrossed *pkIs1641*), *sir-2.1*(O/E)-1X (one-time outcrossed *pkIs1642*). See Table S2C for a repeat of the lifespan of outcrossed *sir-2.1* control and O/E strains. (H) Graph shown in Figure S2B. Worms were grown on RNAi bacteria for 3 generations. This experiment was done once.
(PDF)

Table S2 Lifespan phenotypes of *hcf-1* and *14-3-3* strains. All survival analyses were performed using SPSS software Kaplan Meier analysis and log-rank test to compute *p*-values. *p*-value<0.05 is considered statistically significant. (A) Graph is shown in Figure 2A. *ftt-2* (*ftt-2*-targeting RNAi construct with overlap to *par-5* sequence), *ftt-2gs* (gene-specific *ftt-2* RNAi). LS = Lifespan. Experiment was carried out at 20°C once. (B) Graph is shown in Figure 2B. *par-5* (*par-5-*targeting RNAi construct with overlap to *ftt-2* sequence), *par-5gs* (gene-specific *par-5* RNAi). Experiment was carried out at 20°C once. (C) *sir-2.1*(wt) and *sir-2.1*(O/E) strains are 1X outcrossed in our lab. Experiment was carried out at 25°C. For Figure S3A-S3C, data from two independent experiments are pooled. ^a^ *p*-value vs. N2+vector, ^b^ *p*-value vs. N2+*ftt-2* RNAi, ^c^ *p*-value vs. *pkIs1641*+vector, ^d^ *p*-value vs. *pkIs1641*+*ftt-2* RNAi. (D) Experiment was carried out at 25°C. For Figure S3D, data from two independent experiments and two double mutant isolates are pooled.
(PDF)

Table S3 List of genes significantly changed in the microarray analyses.
(XLS)

Table S4 Gene Ontology term and promoter regulatory motif analyses. (A) Gene ontology term analysis of genes upregulated in *hcf-1*(-), *sir-2.1*(O/E), and *daf-2*(-). Functionally clustered GO terms are summarized and represented by Enrichment score (ES) (depicting how significantly enriched a group of genes within a gene list is over the whole genome: ES of 1 = p-value 1e^−1^. The higher the ES, the more significantly enriched a functional category). Only GO terms with ES> = 1 are shown. *hcf-1*(-)*/sir-2.1*(O/E)-shared = genes in “a+b” (Figure 3D), *hcf-1*(-)*/sir-2.1*(O/E)/*daf-2*(-)-shared = genes in “a” (Figure 3D), *daf-2*(-) only = genes in “g” (Figure 3D). (B) GO term analysis of genes downregulated in *hcf-1*(-), *sir-2.1*(O/E), and *daf-2*(-). (C) a: BioProspector, b: RSAT, c: Two very similar motifs found by BioProspector and RSAT (reverse complements shown), d: The DAF-16 binding element (DBE) was not among the top overrepresented motifs but the presence of this sequence on the candidate gene promoters was directly searched using RSAT.
(PDF)
