# Supplementary figures and images for: The Evolutionarily Conserved Longevity Determinants HCF-1 and SIR-2.1/SIRT1 Collaborate to Regulate DAF-16/FOXO
Source: PLoS Genet. 2011 Sep 1;7(9):e1002235. doi: 10.1371/journal.pgen.1002235 (PMC3164695; doi:10.1371/journal.pgen.1002235)

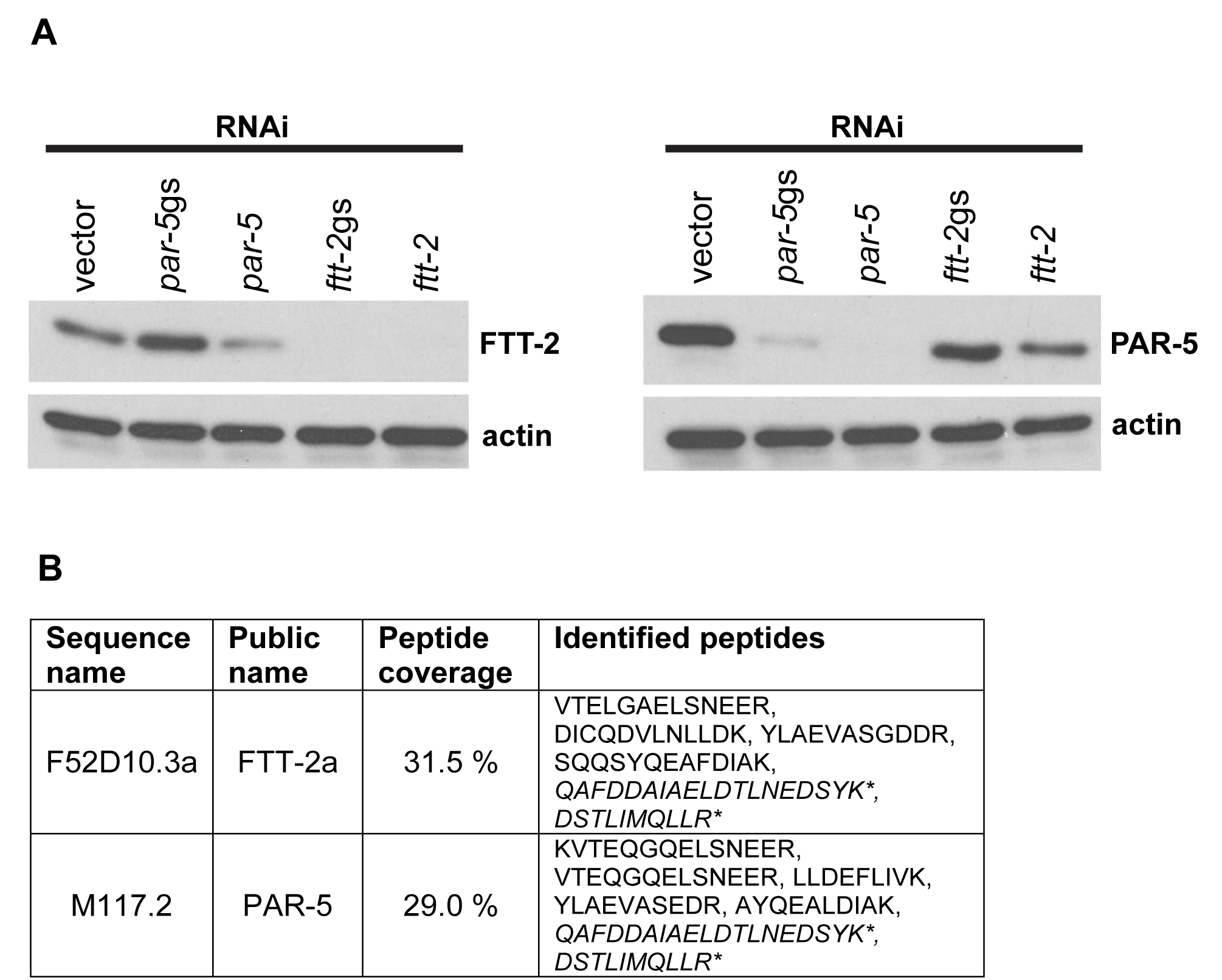

Supplement: Figure S4 — HCF-1 physically interacts with FTT-2 and PAR-5. (A) hcf-1(pk924) worms were grown on plates containing vector control, non-specific (ftt-2 and par-5) or gene-specific (ftt-2gs or par-5gs) 14-3-3 RNAi until young adult stage and protein levels analyzed by western blotting using anti-FTT-2 or anti-PAR-5 antibodies. Actin was used as a loading control. (B) Sequences of the peptides from FTT-2 and PAR-5 proteins, which were identified in the mass spectrometrical analysis of HCF-1::GFP-bound proteins, are listed. *represents peptides that are common to both FTT-2 and PAR-5. (TIF) [file pgen.1002235.s004.tif]

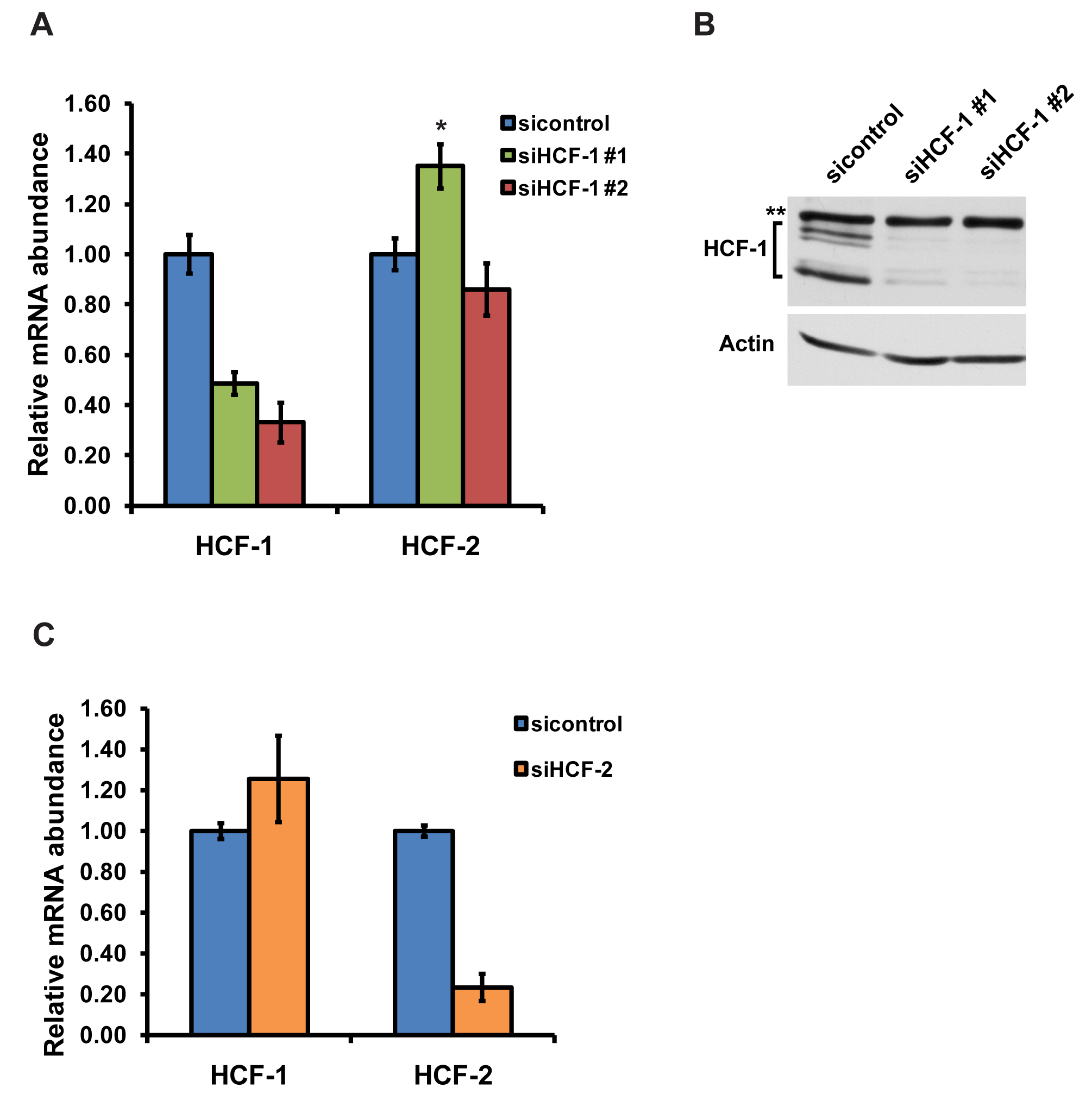

Supplement: Figure S5 — Specific knockdown of HCF-1 and HCF-2 by siRNA. INS-1 cells transfected with HCF-1 (A) or HCF-2 siRNA (B) were analyzed by RT-qPCR and Western blotting. (A) Two different HCF-1 targeting siRNA produced similar effects on FOXO target gene expression. Cells transfected with siHCF-1 #1 exhibited a moderate increase in HCF-2 expression. HCF-2 was not affected by siHCF-1 #2. (B) HCF-1 siRNA substantially reduced HCF-1 protein levels. ** indicates a non-specific band. (C) Knockdown of HCF-2 did not affect HCF-1 expression. Values are normalized to the level of β-actin. The mean normalized mRNA level for each gene in sicontrol treated cells was set to 1. The data represented are pooled from three independent experiments and are represented as mean +/− SEM. * denotes a p-value<0.05. (TIF) [file pgen.1002235.s005.tif]

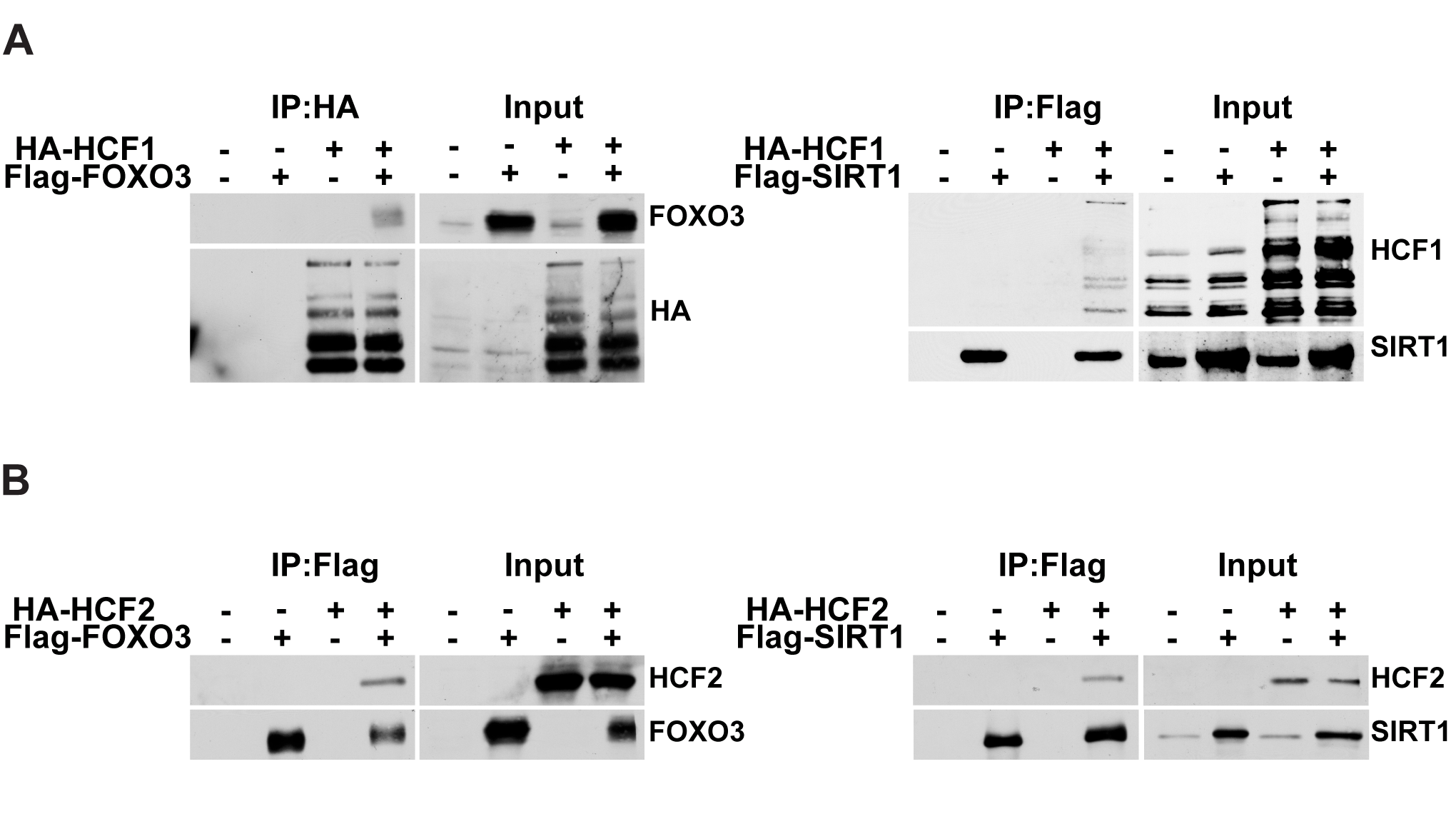

Supplement: Figure S6 — Mammalian HCF homologs physically interact with FOXO3 and SIRT1. HEK293T cells were co-transfected with plasmids encoding HA-HCF1 (A) or HA-HCF2 (B) and either Flag-FOXO3 or Flag-SIRT1. Cell lysates were collected 48 hours later and incubated with either anti-Flag- or anti-HA-conjugated agarose beads. Immunoprecipitated protein complexes were analyzed by western blotting using the indicated antibodies. HCF-1 is known to be proteolytically processed and is detected as multiple bands on SDS-PAGE. (TIF) [file pgen.1002235.s006.tif]
