# Supplementary material for: The Evolutionarily Conserved Longevity Determinants HCF-1 and SIR-2.1/SIRT1 Collaborate to Regulate DAF-16/FOXO
Source: PLoS Genet. 2011 Sep 1;7(9):e1002235. doi: 10.1371/journal.pgen.1002235 (PMC3164695; doi:10.1371/journal.pgen.1002235)
Supplement: Table S1 — Lifespan and oxidative stress phenotypes of hcf-1 and sir-2.1 strains. All survival analyses were done using SPSS software using Kaplan Meier analysis and log-rank test to compute p-values. p-value<0.05 is considered statistically significant. (A) Experiments #1 and #2 were conducted using a lower concentration of bacteria (1X, see Materials and Methods). Experiments #3 and #4 were done on 5X OP50 bacteria. Although the sir-2.1(ok434) mutants have been previously reported to exhibit a slightly shorter lifespan than that of wild-type worms, we observed variable results where the sir-2.1(ok434) mutants tended to live shorter under assaying conditions with lower food (#1&2) and longer on more concentrated bacteria lawns (#3&4). However, whether different bacteria food concentration is the cause of the variability of the sir-2.1 mutant lifespan needs further investigation in the future. Nevertheless, we found that all four independent lines of the double mutants exhibited lifespans similar to that of hcf-1(pk924) single mutant worms, and significantly longer than that of sir-2.1(ok434) mutants. sir-2.1(-) hcf-1(-) (#1–4) represent independent isolates obtained from a cross. * Shown in Figure 1A. Data for each strain, except for sir-2.1(-) hcf-1(-) (#2–4), are pooled from four independent experiments. Data for sir-2.1(-) hcf-1(-) (#2–4) double mutant lines are pooled from two experiments. LS = Lifespan. (B) Experiments were conducted using 5X concentrated OP50 bacteria. hcf-1(pk924);pkIs1642 (#1–5) represent independent isolates obtained from a cross. * Shown in Figure 1B. Data for each strain are pooled from three independent experiments. (C) Survival of worms treated with 6 mM t-BOOH was monitored. Linear Mixed model analysis was used to calculate the averaged percent variation relative to wt. Linear Mixed model analysis allows statistical evaluation of differences between various treatments (mutants) by taking into account the experimental variation. p-value<0.05 is c [file pgen.1002235.s007.pdf]

Table S1A							
***sir-2.1(ok434) hcf-1(pk924)* double mutant analysis in lifespan**							
**Experiment**	**Strain**	**Mean LS + SEM(Days)**	**Total N**	***p*-value vs. N2**	***p*-value vs. *hcf-1(pk924)***	***p*-value vs *sir-2.1(ok434)***	**% effect on N2**
**#1**	N2	14.3 ± 0.2	102		<0.001	0.007	
	hcf-1(pk924)	17.8 ± 0.5	102	<0.001		<0.001	24
	sir-2.1(ok434)	13.3 ± 0.3	100	<0.001	<0.001		-7
	sir-2.1(-) *hcf-1*(-) (#1)	17.0 ± 0.8	51	<0.001	0.906	<0.001	19
	daf-16(mgDf47)	9.4 ± 0.1	102	<0.001	<0.001	<0.001	-34
	daf-16(-);*hcf-1*(-)	8.7 ± 0.2	92	<0.001	<0.001	<0.001	-39
**#2**	N2	13.9 ± 0.2	102		<0.001	0.001	
	hcf-1(pk924)	17.5 ± 0.4	114	<0.001		<0.001	26
	sir-2.1(ok434)	12.8 ± 0.2	99	0.001	<0.001		-8
	sir-2.1(-) *hcf-1*(-) (#1)	18.0 ± 0.5	104	<0.001	<0.001	<0.001	30
	daf-16(mgDf47)	9.2 ± 0.1	100	<0.001	<0.001	<0.001	-34
	daf-16(-);*hcf-1*(-)	8.6 ± 0.2	98	<0.001	0.038	<0.001	-38
**#3**	N2	14.4 ± 0.2	102		<0.001	<0.001	
	hcf-1(pk924)	19.7 ± 0.5	95	<0.001		<0.001	37
	sir-2.1(ok434)	15.6 ± 0.3	100	<0.001	<0.001		8
	sir-2.1(-) *hcf-1*(-) (#1)	20.6 ± 0.8	89	<0.001	0.002	<0.001	43
	sir-2.1(-) *hcf-1*(-) (#2)	18.3 ± 0.5	100	<0.001	0.088	<0.001	27
	sir-2.1(-) *hcf-1*(-) (#3)	21.2 ± 0.7	100	<0.001	0.001	<0.001	47
	sir-2.1(-) *hcf-1*(-) (#4)	20.4 ± 0.6	101	<0.001	0.015	<0.001	42
	daf-16(mgDf47)	9.1 ± 0.1	99	<0.001	<0.001	<0.001	-37
	daf-16(-);*hcf-1*(-)	8.9 ± 0.1	97	<0.001	<0.001	<0.001	-38
**#4**	N2	14.5 ± 0.2	95		<0.001	<0.001	
	hcf-1(pk924)	18.0 ± 0.3	95	<0.001		<0.001	24
	sir-2.1(ok434)	15.3 ± 0.5	92	<0.001	<0.001		6
	sir-2.1(-) *hcf-1*(-) (#1)	17.4 ± 0.2	92	<0.001	0.859	<0.001	19
	sir-2.1(-) *hcf-1*(-) (#2)	18.8 ± 0.4	97	<0.001	0.569	<0.001	20
	sir-2.1(-) *hcf-1*(-) (#3)	19.3 ± 0.3	71	<0.001	0.056	<0.001	30
	sir-2.1(-) *hcf-1*(-) (#4)	18.2 ± 0.2	105	<0.001	0.002	<0.001	34
	daf-16(mgDf47)	9.2 ± 0.1	106	<0.001	<0.001	<0.001	-37
	daf-16(-);*hcf-1*(-)	10.0 ± 0.1	94	<0.001	<0.001	<0.001	-31
**Kaplan Meier analysis - experiments pooled**							
**Experiment**	**Strain**	**Mean LS + SEM(Days)**	**Total N**	***p*-value vs. N2**	***p*-value vs. *hcf-1(pk924)***	***p*-value vs *sir-2.1(ok434)***	**% effect on N2**
**#1-4 pooled**	N2	14.3 ± 0.1	400		<0.001	0.012	
**Figure 1A**	*hcf-1(pk924)*	18.2 ± 0.2	406	<0.001		<0.001	28
	sir-2.1(ok434)	14.2 ± 0.1	390	0.012	<0.001		0
	sir-2.1(-) *hcf-1*(-) (#1)	18.4 ± 0.3	336	<0.001	0.004	<0.001	28
	sir-2.1(-) *hcf-1*(-) (#2)*	17.9 ± 0.3	196	<0.001	0.556	<0.001	25
	sir-2.1(-) *hcf-1*(-) (#3)	20.2 ± 0.5	170	<0.001	<0.001	<0.001	41
	sir-2.1(-) *hcf-1*(-) (#4)	19.9 ± 0.4	205	<0.001	<0.001	0.001	39
	daf-16(mgDf47)	9.2 ± 0.1	406	<0.001	<0.001	<0.001	-35
	daf-16(-);*hcf-1*(-)	9.1 ± 0.1	380	<0.001	<0.001	<0.001	-37

Table S1B

***pkIs1642[sir-2.1*(O/E)*]* and *hcf-1(pk924)* epistasis analysis in lifespan**

Experiment	Strain	Mean LS + SEM(Days)	Total N	*p*-value vs. *hcf-1(pk924)*	*p*-value vs. *pkIs1641*	*p*-value vs *pkIs1642*	% effect on *pkIs1641*
**#1**	N2	15.4 ± 0.2	94	<0.001	0.008	<0.001	4
	hcf-1(*pk924)*	18.4 ± 0.4	101		<0.001	0.002	25
	pkIs1641[sir-2.1(wt)*]*	14.7 ± 0.2	94	<0.001		<0.001	
	pkIs1642[sir-2.1(O/E)*]*	17.5 ± 0.2	104	0.002	<0.001		19
	hcf-1(pk924);pkIs1642 (#1)	17.5 ± 0.4	88	0.122	<0.001	0.584	19
	hcf-1(pk924);pkIs1642 (#2)	19.1 ± 0.4	69	0.401	<0.001	<0.001	30
	hcf-1(pk924);pkIs1642 (#3)	16.2 ± 0.3	110	<0.001	<0.001	0.014	10
	hcf-1(pk924);pkIs1642 (#4)	17.0 ± 0.4	88	0.004	<0.001	0.899	15
	hcf-1(pk924);pkIs1642 (#5)	17.8 ± 0.3	103	0.058	<0.001	0.087	21
	daf-16(mgDf50);*pkIs1642*	8.5 ± 0.1	97	<0.001	<0.001	<0.001	-42
**#2**	N2	15.1 ± 0.1	99	<0.001	0.428	<0.001	1
	hcf-1(*pk924)*	18.9 ± 0.3	96		<0.001	0.001	26
	pkIs1641[sir-2.1(wt)*]*	15.0 ± 0.3	98	<0.001		<0.001	
	pkIs1642[sir-2.1(O/E)*]*	17.3 ± 0.3	101	0.001	<0.001		15
	hcf-1(pk924);pkIs1642 (#1)	16.7 ± 0.6	58	0.007	<0.001	0.971	11
	hcf-1(pk924);pkIs1642 (#2)	19.1 ± 0.4	58	0.427	<0.001	<0.001	27
	hcf-1(pk924);pkIs1642 (#3)	15.9 ± 0.4	104	<0.001	<0.001	0.002	6
	hcf-1(pk924);pkIs1642 (#4)	16.5 ± 0.4	65	<0.001	0.002	0.056	10
	hcf-1(pk924);pkIs1642 (#5)	17.3 ± 0.3	95	<0.001	<0.001	0.982	15
	daf-16(mgDf50);*pkIs1642*	8.5 ± 0.1	97	<0.001	<0.001	<0.001	-43
**#3**	N2	15.0 ± 0.2	98	<0.001	0.727	0.007	0
	hcf-1(*pk924)*	18.9 ± 0.3	100		<0.001	<0.001	28
	pkIs1641[sir-2.1(wt)*]*	15.4 ± 0.2	102	<0.001		<0.001	
	pkIs1642[sir-2.1(O/E)*]*	18.1 ± 0.3	94	<0.001	<0.001		17
	hcf-1(pk924);pkIs1642 (#1)	17.1 ± 0.4	80	0.007	<0.001	0.573	13
	hcf-1(pk924);pkIs1642 (#2)	18.4 ± 0.5	80	0.087	<0.001	<0.001	25
	hcf-1(pk924);pkIs1642 (#3)	15.5 ± 0.3	107	<0.001	<0.001	<0.001	5
	hcf-1(pk924);pkIs1642 (#4)	17.5 ± 0.3	96	<0.001	<0.002	<0.001	9
	hcf-1(pk924);pkIs1642 (#5)	17.5 ± 0.3	96	<0.001	<0.001	0.924	16
	daf-16(mgDf50);*pkIs1642*	9.1 ± 0.1	87	<0.001	<0.001	<0.001	-42

Kaplan Meier analysis - experiments pooled

Experiment	Strain	Mean LS + SEM(Days)	Total N	*p*-value vs. *hcf-1(pk924)*	*p*-value vs. *pkIs1641*	*p*-value vs *pkIs1642*	% effect on *pkIs1641*
**#1-3 pooled**	N2	15.1 ± 0.1	292	<0.001	0.727	0.007	0
**Figure 1B**	*hcf-1*(*pk924)*	19.4 ± 0.2	305		<0.001	<0.001	28
	pkIs1641[sir-2.1(wt)*]*	15.1 ± 0.1	294	<0.001		<0.001	
	pkIs1642[sir-2.1(O/E)*]*	17.6 ± 0.2	299	<0.001	<0.001		17
	hcf-1(pk924);pkIs1642 (#1)	17.1 ± 0.3	226	<0.001	<0.001	0.573	13
	hcf-1(pk924);pkIs1642 (#2)*	18.9 ± 0.2	240	0.087	<0.001	<0.001	25
	hcf-1(pk924);pkIs1642 (#3)	15.9 ± 0.2	321	<0.001	<0.001	<0.001	5
	hcf-1(pk924);pkIs1642 (#4)	16.5 ± 0.2	253	<0.001	<0.001	<0.001	9
	hcf-1(pk924);pkIs1642 (#5)	17.5 ± 0.2	294	<0.001	<0.001	<0.001	16
	daf-16(mgDf50);*pkIs1642*	8.7 ± 0.1	285	<0.001	<0.001	0.924	-42

Table S1C

***sir-2.1(ok434)* and *hcf-1(pk924)* epistasis analysis in *t*-Butyl hydroperoxide**

Kaplan Meier analysis

**Experiment**	**Strain**	**Mean survival + SEM(Hrs)**	**Total N**	**% effect on N2**
**#1**	N2	26.2 ± 0.6	127	
	hcf-1(pk924)	41.8 ± 1.2	105	59.8
	sir-2.1(ok434)	19.0 ± 0.5	109	-27.2
	sir-2.1(-) *hcf-1*(-) (#1)	43.4 ± 1.2	105	66.1
	sir-2.1(-) *hcf-1*(-) (#2)	40.2 ± 1.3	107	53.5
**#2**	N2	24.9 ± 0.9	113	
	hcf-1(pk924)	58.7 ± 2.0	108	136.3
	sir-2.1(ok434)	23.9 ± 1.1	103	-3.7
	sir-2.1(-) *hcf-1*(-) (#1)	73.2 ± 2.7	88	194.5
	sir-2.1(-) *hcf-1*(-) (#2)	55.0 ± 2.1	114	121.4

Linear Mixed model analysis

**#1, 2 pooled**	**Strain**	**Averaged % variation compared to wt ± SEM**	**Total N**	***p*-value vs. *sir-2.1(-) hcf1(-)***
**Figure S1A**	*sir-2.1(ok434)*	-15.5 ± 43.1	212	0.035
	hcf-1(pk924)	98.0 ± 48.9	213	0.715
	sir-2.1(-) *hcf-1*(-)	108.9 ± 46.3	414	

Kaplan Meier analysis - experiments and double mutants pooled

**Experiment**	**Strain**	**Mean survival + SEM(Hrs)**	**Total N**	***p*-value vs. N2**	***p*-value vs. *hcf-1(pk924)***	**% effect on N2**
**#1, 2 pooled**	N2	25.5 ± 0.5	240		<0.001	
**Figure 1C**	*hcf-1(pk924)*	50.1 ± 1.3	212	<0.001		96.0
	sir-2.1(ok434)	21.4 ± 0.6	213	<0.001	<0.001	-16.4
	sir-2.1(-) *hcf-1*(-)	52.2 ± 1.1	414	<0.001	0.193	104.3

Table S1D					
***pkIs1642[sir-2.1*(O/E)] and *hcf-1(pk924)* epistasis analysis in *t*-Butyl hydroperoxide**					
**Kaplan Meier analysis**					
**Experiment**	**Strain**	**Mean survival + SEM(Hrs)**	**Total N**	**% effect on N2**	**% effect on *pkIs1641***
**#1**	N2	26.2 ± 0.6	127		11.4
	pkIs1641[sir-2.1(wt)*]*	23.5 ± 0.6	100	-10.2	
	hcf-1(*pk924)*	41.8 ± 1.2	105	59.8	32.0
	pkIs1642[sir-2.1(O/E)*]*	31.0 ± 0.4	118	18.5	78.0
	hcf-1(pk924);pkIs1642 (#1)	32.3 ± 0.6	115	23.4	37.5
	hcf-1(pk924);pkIs1642 (#2)	43.0 ± 1.1	108	64.3	83.0
**#2**	N2	24.9 ± 0.9	113		13.3
	pkIs1641[sir-2.1(wt)*]*	21.9 ± 0.7	101	-11.8	
	hcf-1(*pk924)*	58.7 ± 2.0	108	136.3	167.7
	pkIs1642[sir-2.1(O/E)*]*	52.3 ± 1.2	109	110.4	138.4
	hcf-1(pk924);pkIs1642 (#1)	46.2 ± 1.2	103	86.1	110.8
	hcf-1(pk924);pkIs1642 (#2)	68.6 ± 1.7	102	176.0	212.7
**Linear Mixed model analysis**					
**#1, 2 pooled**	**Strain**	**Averaged % variation compared to wt ± SEM**	**Averaged % variation compared to *pkIs1641* ± SEM**	**Total N**	***p*-value vs. *hcf-1(pk924); pkIs1642* (wt / *pkIs1641)***
**Figure S1B**	*pkIs1642[sir-2.1*(O/E)*]*	64.4 ± 50.1	85.2 ± 57.9	227	0.557 / 0.560
	hcf-1(pk924)	98.0 ± 47.9	122.9 ± 55.5	213	0.782 / 0.784
	hcf-1(pk924);pkIs1642	87.4 ± 43.4	111.0 ± 50.4	428	

Kaplan Meier analysis - experiments and double mutants pooled						
**Experiment**	**Strain**	**Mean survival + SEM(Hrs)**	**Total N**	***p*-value vs. N2**	***p*-value vs. *hcf-1(pk924)***	**% effect on N2**
**#1, 2 pooled**	N2	25.5 ± 0.5	240		<0.001	
**Figure 1D**	*pkIs1641[sir-2.1*(wt)*]*	22.7 ± 0.5	201	<0.001	<0.001	-11.1
	hcf-1(*pk924)*	50.1 ± 1.3	213	<0.001		96.0
	pkIs1642[sir-2.1(O/E)*]*	41.3 ± 1.0	227	<0.001	<0.001	61.5
	hcf-1(pk924);pkIs1642	47.0 ± 0.9	428	<0.001	0.093	83.9

Table S1E

***sir-2.1(ok434)* and *hcf-1(pk924)* epistasis analysis in paraquat**

Kaplan Meier analysis

Experiment	Strain	Mean survival + SEM(Hrs)	Total N	% effect on N2
**#1**	N2	21.0 ± 1.2	156	
	hcf-1(pk924)	35.9 ± 1.4	177	71.0
	sir-2.1(ok434)	18.6 ± 1.3	193	-11.1
	sir-2.1(-) *hcf-1*(-) (#1)	37.3 ± 1.8	152	77.8
	sir-2.1(-) *hcf-1*(-) (# 2)	29.3 ± 2.0	135	39.8
	sir-2.1(-) *hcf-1*(-) (# 3)	34.9 ± 1.7	157	66.2
	sir-2.1(-) *hcf-1*(-) (# 4)	36.5 ± 1.5	183	74.0
**#2**	N2	33.5 ± 1.1	281	
	hcf-1(pk924)	44.6 ± 1.0	277	33.2
	sir-2.1(ok434)	26.9 ± 1.1	295	-19.7
	sir-2.1(-) *hcf-1*(-) (# 1)	30.4 ± 1.9	116	-9.2
	sir-2.1(-) *hcf-1*(-) (# 2)	39.4 ± 1.3	303	17.6
	sir-2.1(-) *hcf-1*(-) (# 3)	40.9 ± 2.0	129	22.0
	sir-2.1(-) *hcf-1*(-) (# 4)	40.3 ± 0.8	549	20.3

Linear Mixed model analysis

#1, 2 pooled	Strain	Averaged % variation compared to wt ± SEM	Total N	*p*-value vs. ***sir-2.1(-) hcf1(-)***
**Figure S1C**	*sir-2.1(ok434)*	-15.4 ± 24.4	488	0.007
	hcf-1(pk924)	52.1 ± 26.3	454	0.296
	sir-2.1(-) *hcf-1*(-)	38.6 ± 22.1	1725	

Kaplan Meier analysis - experiments and double mutants pooled

Experiment	Strain	Mean survival + SEM(Hrs)	Total N	*p*-value vs. N2	*p*-value vs. ***hcf-1(pk924)***	% effect on N2
**#1,2 pooled**	N2	21.0 ± 1.2	295		<0.001	
**Figure 1E**	*hcf-1(pk924)*	35.9 ± 1.4	314	0.017		47.5
	sir-2.1(ok434)	18.6 ± 1.3	339	<0.001	<0.001	-17.4
	sir-2.1(-) *hcf-1*(-)	37.3 ± 1.8	1079	<0.001	0.686	36.7

Table S1F

***pkIs1642[sir-2.1*(O/E)] and *hcf-1(pk924)* epistasis analysis in paraquat**

Kaplan Meier analysis

Experiment	Strain	Mean survival + SEM(Hrs)	Total N	% effect on N2	% effect on *pkIs1641*
#1	N2	4.8 ± 0.1	114		-8.4
	pkIs1641[sir-2.1(wt)*]*	5.2 ± 0.2	109	9.2	
	hcf-1(*pk924)*	12.3 ± 0.7	109	157.8	136.1
	pkIs1642[sir-2.1(O/E)*]*	13.0 ± 0.5	115	171.5	148.6
	hcf-1(pk924); pkIs1642 (#1)	9.2 ± 0.6	98	93.7	77.4
	hcf-1(pk924);pkIs1642 (#2)	18.8 ± 0.7	109	294.7	261.4
#2	N2	5.5 ± 0.2	116		-12.3
	pkIs1641[sir-2.1(wt)*]*	6.3 ± 0.3	117	14.1	
	hcf-1(*pk924)*	9.5 ± 0.4	120	71.0	49.9
	pkIs1642[sir-2.1(O/E)*]*	10.3 ± 0.5	115	84.8	62.0
	hcf-1(pk924);pkIs1642 (#1)	8.5 ± 0.5	110	53.3	34.4
	hcf-1(pk924);pkIs1642 (#2)	18.9 ± 0.6	115	240.3	198.4

Linear Mixed model analysis

#1,2 pooled	Strain	Averaged % variation compared to wt ± SEM	Averaged % variation compared to *pkIs1641* ± SEM	Total N	*p*-value vs. *hcf-1(pk924); pkIs1642* (wt / *pkIs1641)*
Figure S1D	*pkIs1642[sir-2.1*(O/E)*]*	128.2 ± 73.9	105.3 ± 68.0	230	0.664 / 0.581
	hcf-1(pk924)	114.4 ± 73.9	93.0 ± 67.9	229	0.569 / 0.675
	hcf-1(pk924);pkIs1642	170.5 ± 52.3	142.9 ± 48.1	432	

Kaplan Meier analysis - experiments and double mutants pooled

Experiment	Strain	Mean survival + SEM(Hrs)	Total N	*p*-value vs. N2	*p*-value vs. *hcf-1(pk924)*	% effect on N2
#1, 2 pooled	N2	4.8 ± 0.1	114		<0.001	
Figure 1F	*pkIs1641[sir-2.1*(wt)*]*	5.2 ± 0.2	109	0.105	<0.001	9.2
	hcf-1(*pk924)*	12.3 ± 0.7	109	<0.001		157.8
	pkIs1642[sir-2.1(O/E)*]*	13.0 ± 0.5	115	<0.001	0.831	171.5
	hcf-1(pk924); pkIs1642	14.3 ± 0.6	207	<0.001	0.116	199.6

Table S1G						
**Non-outcrossed and outcrossed *pkIs1642 [sir-2.1*(O/E)*]* lifespan analysis**						
**Strain**	**Mean LS + SEM(Days)**	**Total N**	***p*-value vs. N2**	***p*-value vs. *sir-2.1*(wt)**	***p*-value vs *sir-2.1*(wt)-1X**	**% effect on N2**
N2	14.9 ± 0.1	97		0.832	0.003	
pkIs1641[sir-2.1(wt)*]*	14.7 ± 0.2	93	0.832		0.004	-2
pkIs1642[sir-2.1(O/E)*]*	17.1 ± 0.3	97	<0.001	<0.001	<0.001	14
sir-2.1(wt)-1X	14.3 ± 0.1	100	0.003	0.004		-4
sir-2.1(O/E)-1X	17.2 ± 0.2	95	<0.001	<0.001	<0.001	16

Table S1H						
***sir-2.1* RNAi knockdown lifespan analysis**						
**Strain + RNAi**	**Mean LS + SEM(Days)**	**Total N**	***p*-value vs. N2**	***p*-value vs. *sir-2.1*(wt) + *ctrl* RNAi**	***p*-value vs *sir-2.1*(wt) + *sir-2.1* RNAi**	**% effect on N2+ *ctrl***
N2 + *ctrl*	13.8 ± 0.3	95		0.200	0.314	
N2 + *sir-2.1*	13.7 ± 0.2	96	0.144	0.897	0.669	-1
sir-2.1(wt) + *ctrl*	13.7 ± 0.2	94	0.200		0.767	-1
sir-2.1(wt) + *sir-2.1*	13.8 ± 0.2	87	0.314	0.767		0
sir-2.1(O/E) + *ctrl*	16.1 ± 0.3	96	<0.001	<0.001	<0.001	16
sir-2.1(O/E) + *sir-2.1*	14.3 ± 0.3	94	0.192	0.009	0.020	3
