# Supplementary material for: The Evolutionarily Conserved Longevity Determinants HCF-1 and SIR-2.1/SIRT1 Collaborate to Regulate DAF-16/FOXO
Source: PLoS Genet. 2011 Sep 1;7(9):e1002235. doi: 10.1371/journal.pgen.1002235 (PMC3164695; doi:10.1371/journal.pgen.1002235)
Supplement: Table S2 — Lifespan phenotypes of hcf-1 and 14-3-3 strains. All survival analyses were performed using SPSS software Kaplan Meier analysis and log-rank test to compute p-values. p-value<0.05 is considered statistically significant. (A) Graph is shown in Figure 2A. ftt-2 (ftt-2-targeting RNAi construct with overlap to par-5 sequence), ftt-2gs (gene-specific ftt-2 RNAi). LS = Lifespan. Experiment was carried out at 20°C once. (B) Graph is shown in Figure 2B. par-5 (par-5-targeting RNAi construct with overlap to ftt-2 sequence), par-5gs (gene-specific par-5 RNAi). Experiment was carried out at 20°C once. (C) sir-2.1(wt) and sir-2.1(O/E) strains are 1X outcrossed in our lab. Experiment was carried out at 25°C. For Figure S3A-S3C, data from two independent experiments are pooled. a p-value vs. N2+vector, b p-value vs. N2+ftt-2 RNAi, c p-value vs. pkIs1641+vector, d p-value vs. pkIs1641+ftt-2 RNAi. (D) Experiment was carried out at 25°C. For Figure S3D, data from two independent experiments and two double mutant isolates are pooled. (PDF) [file pgen.1002235.s008.pdf]

**Table S2A**						
***ftt-2* or *ftt-2* gene specific RNAi and *hcf-1(pk924)* epistasis analysis in lifespan**						
**Strain**	**RNAi**	**Mean LS + SEM(Days)**	**Total N**	**% lifespan extension by *hcf-1(-)*+RNAi vs. corresponding N2+RNAi**	***p*-value vs. N2+vector**	***p*-value vs. *hcf-1(-)* + vector RNAi**
N2	vector	18.6 ± 0.2	90			<0.001
	ftt-2	14.0 ± 0.3	95		<0.001	<0.001
	ftt-2gs	17.0 ± 0.2	89		<0.001	<0.001
	daf-16	11.7 ± 0.2	93		<0.001	<0.001
hcf-1(pk924)	vector	22.5 ± 0.4	98	21.0	<0.001	
	ftt-2	12.4 ± 0.3	97	-11.9	<0.001	<0.001
	ftt-2gs	20.5 ± 0.4	106	20.4	<0.001	<0.001
	daf-16	11.8 ± 0.1	109	0.7	<0.001	<0.001

**Table S2B**						
***par-5* or *par-5* gene specific RNAi and *hcf-1(pk924)* epistasis analysis in lifespan**						
**Strain**	**RNAi**	**Mean LS + SEM(Days)**	**Total N**	**% lifespan extension by *hcf-1(-)* +RNAi vs. corresponding N2+RNAi**	***p*-value vs. N2+vector**	***p*-value vs. *hcf-1(-)* + vector RNAi**
N2	vector	18.6 ± 0.2	120			<0.001
	par-5	13.3 ± 0.2	114		<0.001	<0.001
	par-5gs	18.6 ± 0.2	115		<0.001	<0.001
	daf-16	11.1 ± 0.1	103		<0.001	<0.001
hcf-1(pk924)	vector	25.0 ± 0.6	97	34.6	<0.001	
	par-5	10.8 ± 0.2	97	-18.6	<0.001	<0.001
	par-5gs	25.0 ± 0.5	92	34.3	<0.001	0.645
	daf-16	10.7 ± 0.2	91	-3.6	<0.001	<0.001

Table S2C							
ftt-2 **RNAi and** ***hcf-1(pk924), sir-2.1***(**O/E**), **or** ***daf-2(e1370)*** **epistasis analysis in lifespan**							
**Experiment**	**Strain**	**RNAi**	**Mean LS + SEM(Days)**	**Total N**	**% lifespan extension on vector RNAi**	**% lifespan extension on *ftt-2* RNAi**	***p*-value vs. control+ RNAi**
**#1**	N2	vector	16.7 ± 0.3	103			
		ftt-2	15.8 ± 0.2	100			
	hcf-1(pk924)	vector	18.3 ± 0.3	103	9 (vs. N2)		<0.001[a]
		ftt-2	15.6 ± 0.2	106		-1	0.885[b]
	pkIs1641[sir-2.1(wt)*]*	vector	18.7 ± 0.3	107			
		ftt-2	16.0 ± 0.2	111			
	pkIs1642[sir-2.1(O/E)*]*	vector	21.0 ± 0.3	107	12 (vs. pkIs1641)		<0.001[c]
		ftt-2	16.6 ± 0.3	112		-4	0.050[d]
	daf-2(e1370)	vector	30.3 ± 1.1	100	81 (vs. N2)		<0.001[a]
		ftt-2	26.9 ± 1.2	99		71	<0.001[b]
**#2**	N2	vector	16.8 ± 0.2	104			
		ftt-2	16.3 ± 0.2	108			
	hcf-1(pk924)	vector	18.0 ± 0.3	104	7 (vs. N2)		0.002[a]
		ftt-2	15.6 ± 0.3	102		-4	0.070[b]
	pkIs1641[sir-2.1(wt)*]*	vector	19.0 ± 0.3	107			
		ftt-2	17.1 ± 0.3	105			
	pkIs1642[sir-2.1(O/E)*]*	vector	20.9 ± 0.4	95	10 (vs. pkIs1641)		<0.001[c]
		ftt-2	16.1 ± 0.3	104		-6	0.013[d]
	daf-2(e1370)	vector	26.1 ± 1.0	93	55 (vs. N2)		<0.001[a]
		ftt-2	22.6 ± 0.8	103		38	<0.001[b]
**Kaplan Meier Analysis - experiments pooled**							
**Experiment**	**Strain**	**RNAi**	**Mean LS + SEM(Days)**	**Total N**	**% lifespan extension on vector RNAi**	**% lifespan extension on *ftt-2* RNAi**	***p*-value vs. control+ RNAi**
**#1, 2 pooled**	N2	vector	16.8 ± 0.2	207			
**Figure S3A-C**		*ftt-2*	16.1 ± 0.1	208			
	hcf-1(pk924)	vector	18.1 ± 0.2	207	8 (vs. N2)		<0.001[a]
		ftt-2	15.6 ± 0.2	208		-3	0.139[b]
	pkIs1641[sir-2.1(wt)*]*	vector	18.9 ± 0.2	214			
		ftt-2	16.5 ± 0.2	216			
	pkIs1642[sir-2.1(O/E)*]*	vector	20.9 ± 0.2	202	11 (vs. pkIs1641)		<0.001[c]
		ftt-2	16.3 ± 0.2	216		-1	0.529[d]
	daf-2(e1370)	vector	28.4 ± 0.7	198	69 (vs. N2)		<0.001[a]
		ftt-2	24.5 ± 0.7	199		50	0.007[b]

Table S2D

***ftt-2(n4426)* and *hcf-1(pk924)* epistasis analysis in lifespan**

Experiment	Strain	Mean LS + SEM(Days)	Total N	% lifespan extension vs. N2	% lifespan extension vs. *ftt-2(-)*	*p*-value vs. N2
**#1**	N2	15.8 ± 0.2	107			
	hcf-1(pk924)	22.2 ± 0.4	105	41		<0.001
	ftt-2(n4426)	12.6 ± 0.2	100	-20		<0.001
	hcf-1(-);ftt-2(-) (#1)	18.9 ± 0.4	94	20	50	<0.001
	hcf-1(-);ftt-2(-) (#2)	19.2 ± 0.4	81	22	53	<0.001
**#2**	N2	15.1 ± 0.2	105			
	hcf-1(pk924)	19.8 ± 0.4	104	31		<0.001
	ftt-2(n4426)	14.2 ± 0.2	100	-6		0.002
	hcf-1(-);ftt-2(-) (#1)	18.8 ± 0.3	88	24	32	<0.001
	hcf-1(-);ftt-2(-) (#2)	19.4 ± 0.4	78	28	37	<0.001

Kaplan Meier analysis - experiments pooled

Experiment	Strain	Mean LS + SEM(Days)	Total N	% lifespan extension vs. N2	% lifespan extension vs. *ftt-2(-)*	*p*-value vs. N2
**#1,2 pooled**	N2	15.5 ± 0.1	212			
**Figure S3D**	*hcf-1(pk924)*	21.0 ± 0.3	209	36		<0.001
	ftt-2(n4426)	13.4 ± 0.2	200			<0.001
	hcf-1(-);ftt-2(-)	19.1 ± 0.2	341		43	<0.001
