# Supplementary material for: The Evolutionarily Conserved Longevity Determinants HCF-1 and SIR-2.1/SIRT1 Collaborate to Regulate DAF-16/FOXO
Source: PLoS Genet. 2011 Sep 1;7(9):e1002235. doi: 10.1371/journal.pgen.1002235 (PMC3164695; doi:10.1371/journal.pgen.1002235)
Supplement: Table S4 — Gene Ontology term and promoter regulatory motif analyses. (A) Gene ontology term analysis of genes upregulated in hcf-1(-), sir-2.1(O/E), and daf-2(-). Functionally clustered GO terms are summarized and represented by Enrichment score (ES) (depicting how significantly enriched a group of genes within a gene list is over the whole genome: ES of 1 = p-value 1e−1. The higher the ES, the more significantly enriched a functional category). Only GO terms with ES> = 1 are shown. hcf-1(-)/sir-2.1(O/E)-shared = genes in “a+b” (Figure 3D), hcf-1(-)/sir-2.1(O/E)/daf-2(-)-shared = genes in “a” (Figure 3D), daf-2(-) only = genes in “g” (Figure 3D). (B) GO term analysis of genes downregulated in hcf-1(-), sir-2.1(O/E), and daf-2(-). (C) a: BioProspector, b: RSAT, c: Two very similar motifs found by BioProspector and RSAT (reverse complements shown), d: The DAF-16 binding element (DBE) was not among the top overrepresented motifs but the presence of this sequence on the candidate gene promoters was directly searched using RSAT. (PDF) [file pgen.1002235.s010.pdf]

Table S4A. Enriched GO terms for upregulated genes				
**GO terms**	***hcf-1*(-) / *sir-2.1*(O/E) -shared**	***hcf-1*(-) / *sir-2.1*(O/E) / *daf-2*(-) -shared**	***daf-2* (-) only**	**Biological process**
Determination of adult lifespan	5.25	5.83		Aging
Glutathione S-transferase	2.93	3.24		Detoxification
Alcohol dehydrogenase	2.35	2.52		Detoxification
Short-chain dehydrogenase	2.20	2.49	2.14	Detoxification
Cytochrome P450	2.10	2.54		Detoxification
Antioxidant/ Oxidoreductase	1.99	2.20	1.73	Detoxification
Cofactor metabolic process	1.96	2.37		
Zinc-finger RING, BBOX	1.85	0.99		
Metal ion binding	1.72	1.19		
Carboxylesterase (type B)	1.60	1.65	1.02	Detoxification
Lysosome	1.52			Detoxification
Endopeptidase inhibitor (cysteine, serpin)	1.44	1.66		Detoxification
G-protein signaling inhibitor	1.10			Signaling
Dauer development		1.06		Development
Phenol metabolic process		1.05		Metabolism
Collagen / cuticle			13.44	
Glycoprotein/ signaling			6.27	Development: pharyngeal, embryonic, neuronal
Neuropeptide signaling			2.63	Development: pharyngeal
EF-hand calcium binding (calmodulin)			2.50	Development: body morphology, locomotion, egglaying
Peptidase/proteolysis			2.32	Metabolism
Cytoskeleton organization			2.12	
UDP-glucuronosyl/UDP-glucosyltransferase			1.99	Detoxification
LIM domain			1.95	Development: muscle, neuron
Patched / hedgehog receptor			1.85	Development : growth, locomotion
Amino acid metabolism (degradation)			1.70	Metabolism
EGF-like domain			1.64	Development: growth
Organismal growth			1.53	
Anion transport			1.48	Cellular homeostasis
Ion homeostasis			1.13	Cellular homeostasis
Glycolysis/Gluconeogenesis			1.12	Metabolism
Sulfur amino acid biosynthesis			1.07	Metabolism
Intermediate filament			1.01	Development: body morphogenesis

**Table S4B. Enriched GO terms for downregulated genes**				
**GO terms**	***hcf-1*(-) / *sir-2.1*(O/E) -shared**	***hcf-1*(-) / *sir-2.1*(O/E) / *daf-2*(-) -shared**	***daf-2* (-) only**	**Biological process**
Glycoprotein/signaling	3.95	3.76		Cellular signaling
Amino acid biosynthesis	3.14	1.84		Metabolism
Collagen/cuticle	3.00			
SaposinB	2.41			Metabolism: lipid degradation
Acid phosphatase	2.22	1.96		Metabolism: digestion (lysosomal)
Peptidase (serine)	2.18			Metabolism: proteolysis (lysosomal)
Determination of adult lifespan	2.15	2.54		Aging
Lysozyme	1.99	2.24		Immunity
Ribosomal proteins	1.80	2.24	9.96	Translation
Fatty acid biosynthesis	1.60	1.62		Metabolism
Peptidase/proteolysis (C1)	1.51	1.05		Metabolism
UDP-glucuronosyltransferase	1.50	1.76		Detoxification
N-acetyltransferase	1.44			Detoxification
EGF-like domain	1.40			Signaling
C-type lectin	1.31	1.00		Immunity
Carboxylesterase (type B)	1.27			Detoxification
Peptidase/proteolysis (A1)	1.19	1.36		Metabolism
Iron/sulphur/FAD binding	1.18			
FAD-binding	1.15			
Organismal growth (positive)	1.13		14.18	
Peptidase/proteolysis		1.57		
Metal ion transport/binding		1.08		
Larval development			20.93	Development
Hermaphrodite genitalia devel.			7.89	Development
Mitochondrial			6.47	Protein translation/transport
Translation initiation			4.67	Protein biosynthesis
Protein folding/UPR(Chaperone)			4.54	Protein homeostasis
Aminoacyl-tRNA synthetase			4.25	Protein biosynthesis
Protein transport			4.07	Vesicular biogenesis/transport
Endomembrane system			3.59	Potein transport: ER, Golgi, nuclear
Protein targeting/mitochondria			2.99	Protein transport
Organelle lumen			2.94	
WD40 repeat			2.77	
RNA polymerase			2.49	Transcription
Nuclear pore complex			2.42	Nuclear transport
Proteasome core complex			2.33	Protein degradation
Small ribonucleoprotein			2.18	RNA splicing/degradation. Development
Chromatin/Histones			2.18	Chromatin structure/DNA replication
Proteasome component			1.95	Protein degradation
Organismal growth (negative)			1.95	
Golgi apparatus			1.88	Vesicle /transport
Chaperones			1.77	Protein folding
Tetratricopeptide repeat			1.69	Embryonic development
RNA metabolism/processing			1.68	RNA Splicing
Ubq-dependent proteolysis			1.58	Protein degradation
Nuclear migration			1.40	Mitosis / cytokinesis
Cell division			1.39	
Ubiquitin-ligase			1.38	Protein degradation
Embryonic development			1.32	
Heat shock protein DnaJ			1.30	UPR, protein homeostasis
Axon/ neuron projection			1.26	Development
RNA-recognition motif			1.24	RNA processing
Cell division/cyclins			1.23	
cis-trans isomerase			1.18	Protein folding/ immunity
Signal peptide processing			1.15	Protein processing / maturation
AAA+ ABC transporter			1.08	Detoxifications

Table S4C. Enriched promoter regulatory motifs.				
***hcf-1*(-) / *sir-2.1*(O/E) / *daf-2*(-) – shared genes (693 genes)**		***daf-2*(-) – specific genes (1750 genes)**		
**Motif**	**% of genes with motif**	**Motif**	**% of genes with motif**	**Known consensus transcription factor binding site**
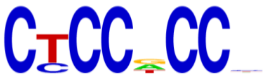 **a**	48%	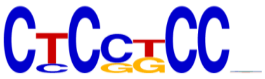 **a**	70%	Mammalian SP1 p-value<0.001
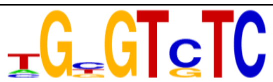 **a,c**	45%	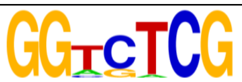 **a,c**	66%	Mammalian Fhl p-value<0.001
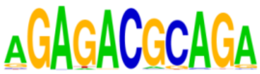 **b,c**	16%	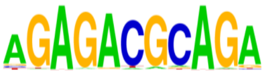 **b,c**	13%	
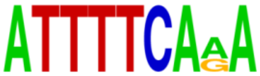 **b**	68%	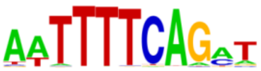 **b**	87%	Mammalian Oct-1 p-value<0.001
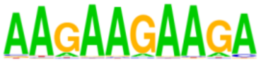 **b**	68%	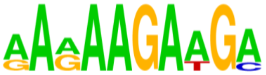 **b**	42%	
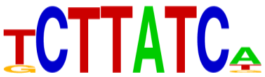 **a,b**	54%	**Not enriched**		(1) Mammalian GATA (*C. elegans elt-3,5,6)* p-value<0.0001<br><br>(2) Mammalian Evi-1 (*C. elegans egl-43),* p-value<0.0001
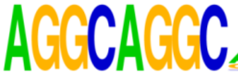 **b**	10%	**Not enriched**		
**Not enriched**		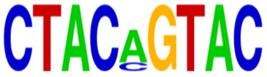 **b**	10%	
**DBE: T(G/A)TTTAC[d]**	51%	**DBE: T(G/A)TTTAC[d]**	45%	
